# Brain microglia serve as a persistent HIV reservoir despite durable antiretroviral therapy

**DOI:** 10.1172/JCI167417

**Published:** 2023-06-15

**Authors:** Yuyang Tang, Antoine Chaillon, Sara Gianella, Lilly M. Wong, Dajiang Li, Theresa L. Simermeyer, Magali Porrachia, Caroline Ignacio, Brendon Woodworth, Daniel Zhong, Jiayi Du, Eduardo de la Parra Polina, Jennifer Kirchherr, Brigitte Allard, Matthew L. Clohosey, Matt Moeser, Amy L. Sondgeroth, Gregory D. Whitehill, Vidisha Singh, Amir Dashti, Davey M. Smith, Joseph J. Eron, Katherine J. Bar, Ann Chahroudi, Sarah B. Joseph, Nancie M. Archin, David M. Margolis, Guochun Jiang

**Affiliations:** 1 University of North Carolina (UNC) HIV Cure Center, and; 2 Division of Infectious Diseases, Department of Medicine, University of North Carolina at Chapel Hill, Chapel Hill, North Carolina, USA.; 3 Department of Medicine, UCSD, La Jolla, California, USA.; 4 UNC Lineberger Comprehensive Cancer Center, University of North Carolina at Chapel Hill, Chapel Hill, Chapel Hill, North Carolina, USA.; 5 Department of Medicine, University of Pennsylvania, Philadelphia, Pennsylvania, USA.; 6 Department of Pediatrics, Emory University School of Medicine, Atlanta, Georgia, USA.; 7 Department of Epidemiology, Gillings School of Global Public Health, University of North Carolina at Chapel Hill, North Carolina, USA.; 8 Center for Childhood Infections and Vaccines of Children’s Healthcare of Atlanta and Emory University, Atlanta, Georgia, USA.; 9 Department of Microbiology and Immunology, UNC School of Medicine, University of North Carolina at Chapel Hill, Chapel Hill, North Carolina, USA.; 10 Department of Epidemiology, Gillings School of Global Public Health, University of North Carolina at Chapel Hill, North Carolina, USA.

**Keywords:** AIDS/HIV, Drug therapy

## Abstract

Brain microglia (MG) may serve as a human immunodeficiency virus 1 (HIV) reservoir and ignite rebound viremia following cessation of antiretroviral therapy (ART), but they have yet to be proven to harbor replication-competent HIV. Here, we isolated brain myeloid cells (BrMCs) from nonhuman primates and rapid autopsy of people with HIV (PWH) on ART and sought evidence of persistent viral infection. BrMCs predominantly displayed microglial markers, in which up to 99.9% of the BrMCs were TMEM119^+^ MG. Total and integrated SIV or HIV DNA was detectable in the MG, with low levels of cell-associated viral RNA. Provirus in MG was highly sensitive to epigenetic inhibition. Outgrowth virus from parietal cortex MG in an individual with HIV productively infected both MG and PBMCs. This inducible, replication-competent virus and virus from basal ganglia proviral DNA were closely related but highly divergent from variants in peripheral compartments. Phenotyping studies characterized brain-derived virus as macrophage tropic based on the ability of the virus to infect cells expressing low levels of CD4. The lack of genetic diversity in virus from the brain suggests that this macrophage-tropic lineage quickly colonized brain regions. These data demonstrate that MG harbor replication-competent HIV and serve as a persistent reservoir in the brain.

## Introduction

Efforts to clear HIV infection require a careful assessment of all the tissue reservoirs in which the virus persists despite durable antiretroviral therapy (ART). The CNS is a site with unique biological, immunological, and pharmacological features (1). However, rigorous evidence of viral persistence in the CNS cells of humans on durable suppressive ART is incomplete (2–5). Circulating T cells have been well characterized as the major HIV reservoir in the peripheral blood and may circulate into the CNS, contributing to HIV persistence in the brain (6). Myeloid cells are another major cellular component of the immune system infected by SIV and HIV, and infection may persist in brain myeloid cells (BrMCs) (7–13). Viral rebound in the CNS was reported upon interruption of therapy in the humanized myeloid-only mouse model of HIV latency (11). Replication-competent SIV has also been reported in brain macrophages of ART-suppressed, SIV-infected rhesus macaques (14–16).

Given the obvious challenges in accessing human brain tissue, the HIV reservoir in BrMCs, including long-lived and self-renewing microglia (MG), has not been rigorously studied in people with HIV (PWH) on long-term ART. The lack of physiologically relevant cellular model systems that faithfully replicate persistent HIV infection in BrMCs or MG poses another challenge to the study of these CNS cell types. Immortalized MG cell lines, such as HMC3 and C20, have been used as HIV infection models but have transcriptional profiles distinct from that of primary MG (17), making them less relevant to the study of the biology of HIV persistence in the CNS. Human studies of HIV persistence in the CNS have been limited to the examination of archived postmortem brain tissues. Most studies of HIV reservoirs in brain tissue have lacked confirmation of ART adherence and viral suppression prior to death and may have utilized brain tissues that had begun to degrade prior to collection. Thus, it remains unclear whether human BrMCs, and especially long-lived MG, are latently infected, and if they encode replication-competent HIV despite durable, successful ART.

To address these challenges, we first developed protocols to isolate highly pure populations of BrMCs and MG from the tissues of nonhuman primates (NHPs). We then adapted these protocols to the study of human brain tissues containing large numbers of viable cells after rapid autopsy to explore whether human BrMCs produce replication-competent HIV. We studied rapid-autopsy samples from altruistic PWH on suppressive ART who were enrolled in the “Last Gift” cohort (18, 19), as well as tissue from the National Disease Research Interchange (NDRI). We believe our method for isolating viable BrMCs provides a physiologically relevant platform for studies of the biology of CNS reservoirs and, ultimately, will aid in efforts toward HIV eradication.

## Results

### Development of methods for the selection of viable, purified brain MG following necropsy in ART-suppressed, SIV-infected rhesus macaques.

**Figure 1:**
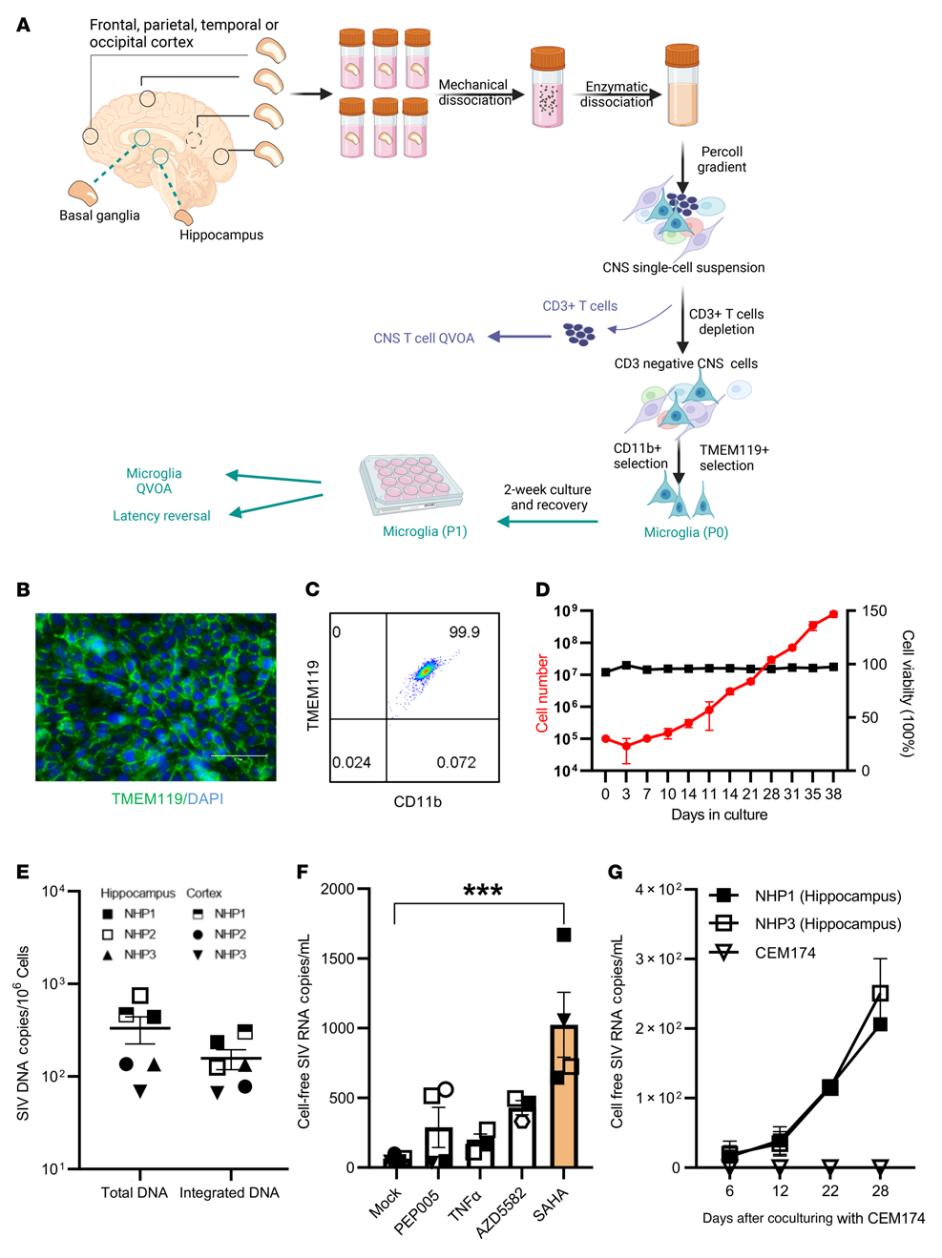
Isolation and characterization of BrMCs and MG from ART-suppressed NHPs and PWH. (**A**) Brain tissue pieces were collected from the indicated brain regions and dissociated by mechanical disruption and enzymatic digestion. A CNS single-cell suspension was generated after Percoll separation. CD3^+^ T cells were positively selected and used for a CNS T cell QVOA. BrMCs and MG were isolated from the CD3^–^ fractions by CD11b^+^ selection and by TMEM119^+^ selection, respectively. For human brains, CD11b^+^ selection was performed to isolate BrMCs. BrMCs or MG at P0 (collected immediately after isolation) were used for purity and phenotype analysis and for RT-qPCR to measure proviral DNA and cell-associated RNA. BrMCs or MG at P1 were cultured 1–2 weeks ex vivo to allow the cells to recover and attach. P1 cells were used for the LRA study and the QVOA. (**B**) MG isolated from ART-suppressed, SIV-infected rhesus macaques were defined by TMEM119 staining (P1 MG) (scale bar: 100 μm) and (**C**) anti-TMEM119/anti-CD11b flow cytometry (P0 MG). (**D**) NHP MG proliferated ex vivo. (**E**) Total and integrated SIV DNA was detectable in isolated P0 MG (*n* = 3). (**F**) SIV RNA was induced in isolated P1 MG 7 days after stimulation by the HDACi SAHA (500 nM), but was poorly induced by the canonical NF-κB agonist PEP005 (12 nM), the noncanonical NF-κB agonist AZD5582 (100 nM), or TNF-α (50 ng/mL). ***P < 0.001 compared with mock treatment, by 1-way ANOVA (*n* = 3). (**G**) SIV RNA was recovered from the supernatant of NHP P1 MG cocultured with CEM174 (*n* = 3). Data are presented as the mean ± SEM.

Four SIV239-infected rhesus macaques (*n* = 4) were studied at the time of necropsy following ART suppression for 101–105 weeks (plasma viremia <60 copies/mL) (Supplemental Table 1; supplemental material available online with this article; https://doi.org/10.1172/JCI167417DS1). As outlined in Figure 1A, the brain tissue pieces from the indicated brain regions were dissociated into a single-cell suspension by performing enzymatic and mechanical dissociation steps and Percoll gradients to remove debris. CD3^+^ T cells were rigorously depleted using anti-CD3 microbeads. BrMCs were isolated from the CD3^–^ cellular fraction after a second selection using CD11b (15), a myeloid cell–surface marker presented on both monocyte-derived macrophages (MDMs) and MG (20–22). To evaluate a proper separation of BrMCs, aliquots of CNS cells during pre- and post-CD11b selection were further analyzed for expression of the myeloid marker CD68. Nearly 31.6% of the preisolated cells were CD68^+^ (Supplemental Figure 1A), whereas more than 99% of these cells were CD68^+^ after CD11b selection, confirming that the isolated cells were highly pure BrMCs (Supplemental Figure 1, B and C).

As MG are self-renewing and long-lived (21, 23–26), these features may allow infected MG to persist during long-term suppressive ART, forming a stable CNS reservoir in the brain. We sought to directly isolate MG from isolated BrMCs. Earlier reports revealed that MDMs and MG can be distinguished by the expression level of CD45 (27, 28). Consistent with previous studies in rodents (29, 30), after selection, 93.5% of CD68^+^ BrMCs were CD45^lo^ MG, while less than 5% of these cells were CD45^hi^ macrophages (Supplemental Figure 1D).

Recently, TMEM119 has been identified as a novel and specific MG surface marker in brain MG in rodent models and in a humanized mouse model (31, 32). We next purified MG from brain tissues of SIV-infected macaques by positive selection of the TMEM119^+^ cells, separating them from other BrMCs. A highly pure population of MG was obtained, in which 99.9% of these cells were CD11b^+^TMEM119^+^ (Figure 1, B and C, and Supplemental Figure 2), indicating that these isolated cells were brain MG. Interestingly, these cells proliferated well ex vivo (Figure 1D), allowing long-term culturing (>9 weeks). These MG were free of detectable T cells, as we detected no CD3E RNA expression in highly sensitive reverse transcription quantitative PCR (RT-qPCR) assays with a limit of detection of 1 CD3^+^ T cell per million CNS cells (Tables 1 and 2). Both total and integrated SIV DNA was detectable in isolated MG (*n* = 3) (Figure 1E). SIV RNA (*n* = 4) was low in isolated cells in culture (average of 63.25 copies/mL), but SIV RNA was inducible by the histone deacetylase inhibitor (HDACi) SAHA (33) (*P* < 0.001, 16-fold increase compared with mock treatment) (Figure 1F). Surprisingly, 7 days after treatment, we found that SIV RNA was poorly induced by NF-κB agonists such as PEP005 (34), AZD5582 (35), or TNF-α (34, 36) (Figure 1F). SIV RNA was gradually released into tissue culture media after coculturing of SAHA-reversed MG with CEM174 cells for up to 28 days (*n* = 2; Figure 1G), indicating a replication-competent reservoir in MG. Together, these data provide proof of concept in the NHP model, with experimental evidence that a highly pure population of brain MG can be isolated and used for viral reservoir studies (12, 15).

### Participant characteristics.

Having established this rigorous MG isolation technique, we sought to examine HIV persistence in pure populations of brain MG isolated from 3 PWH enrolled in the Last Gift program (18, 19) and from 1 individual with HIV from the NDRI (Table 3). Donors 1 and 2 remained aviremic (<30 copies/mL) and were last assayed 6 days prior to their death. Donor 3 was also suppressed on ART but stopped ART 18 days before death with detectable viremia (275 copies/mL) on the day of autopsy. Donor 4 (from NDRI) stopped ART 6 days before death, and plasma viremia data near the time of death were unavailable. The average age of the donors was 63.4 ± 10.8 years, including 2 White males, 1 White female, and 1 White transgender female. The CD4 count ranged from 87–237 cells/μL at the time of death. As intended in the Last Gift cohort, each of these PWH had a terminal disease (Table 3). Four donors without HIV from the NDRI were included as controls. The average age of these donors was 73.5 ± 14.5 years, including 2 White females and 2 White males (Supplemental Table 2).

### Viable MG can be readily isolated from brain tissue after rapid autopsy.

The protocol outlined in Figure 1A was then optimized and applied to isolate human MG from different brain regions of PWH on ART (*n* = 4) and from donors without HIV (*n* = 4). T cells and BrMCs were captured sequentially with antibodies recognizing the surface protein T cell marker CD3 and the pan–myeloid cell marker CD11b. We did not further perform TMEM119 selection for human MG, given the low yield of human TMEM119^+^ MG with the previously reported anti–human TMEM119 extracellular domain antibody (32).

**Figure 2:**
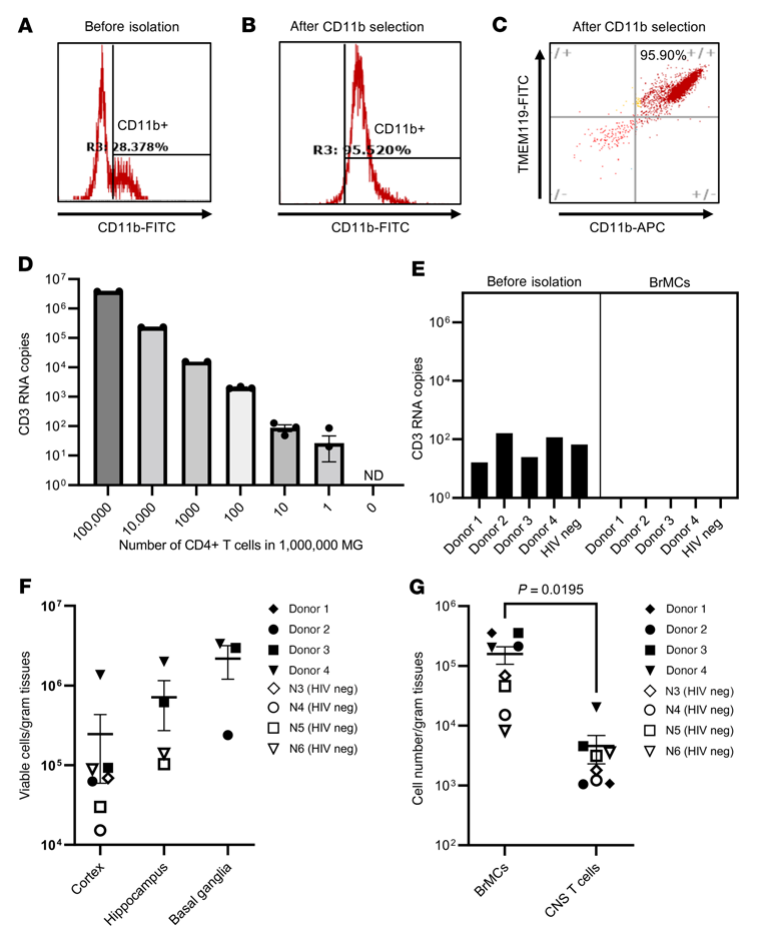
Isolation of highly pure and viable MG from fresh postmortem brain tissues of PWH on ART. Representative FACS plots show the CD11b^+^ cells in a CNS single-cell suspension from the parietal cortex of donor 2 before (**A**) and after (**B**) CD11b selection. (**C**) Greater than 95% of CD11b^+^ BrMCs were TMEM119^+^ MG after enrichment. (**D**) Standard for a highly sensitive CD3 ddPCR assay with a detection limit of 1 CD4^+^ cell per 1,000,000 MG. ND, not detectable. (**E**) CD3 RNA was undetectable in 1 × 10^6^ isolated MG, but was detectable before isolation (*n* = 5). (**F**) MG yields in different brain regions of donor brains with or without HIV (*n* = 7). (**G**) The number of isolated MG was much higher than CNS T cells isolated from the same source of tissues from donors with or without HIV. *P* = 0.0195, by paired Student’s *t* test (*n* = 7). Data are presented as the mean ± SEM. neg, negative.

The purity of isolated BrMCs via CD11b immunoselection was initially examined by immunostaining. We found that approximately 28.38% of CNS cells were CD11b^+^ before BrMC isolation (Figure 2A). After selection, 95.52% of these cells were CD11b^+^ (Figure 2B). To distinguish MG from other BrMCs, we further labeled the cells with CD11b and the MG marker TMEM119. We observed that 95.9% of CD11b^+^ BrMCs also expressed TMEM119 (Figure 2C), indicating that, like in NHP (Supplemental Figure 1D), MG are the major population of BrMCs in human brain tissue (29, 30). Hereafter, we refer to these CD11b^+^ cells as MG but do not exclude the minuscule amount of brain macrophages.

To ensure that isolated MG were free of detectable T cells, we performed highly sensitive CD3 droplet digital PCR (ddPCR) with a limit of detection of 1 CD3^+^ T cell per million MG (Figure 2D). Although 30–240 CD3 RNA copies/million CNS cells were detected before MG isolation, no CD3 was detectable in the purified MG cell populations (Figure 2E).

Among 4 PWH and 4 donors without HIV, 10^5^ to 10^6^ viable MG/gram tissue were recovered from deep brain regions, including the hippocampus and basal ganglia, whereas fewer viable CD11b^+^ MG were isolated from the cortex regions (10^4^ to 10^6^/gram; Figure 2F). We found that the autopsy timeline was critical for the successful isolation of these viable MG. The autopsies were optimal if performed within 6 hours of death, the window of time during which up to 90% of MG are viable. The number may drop quickly to less than 1% if the autopsy is performed more than 48 hours after death (from NDRI) because of brain deterioration, which would render the MG unusable for our study.

Notably, among PWH, the average yield (10^5^ to 10^6^/gram) of MG across different regions was nearly 10 times more than the numbers of MG obtained from the 4 donors without HIV (10^4^ to 10^5^/gram) (Figure 2G). In contrast, the total CNS cell numbers (obtained from Percoll gradient steps) were quite similar between HIV^+^ and HIV^–^ donors (nearly 10^6^ to 10^7^/gram tissue). The increased number of MG detected in the brains of PWH suggested the presence of in vivo MG proliferation and activation in these HIV^+^ donors despite ART. For CNS T cell isolation, the numbers of CD3^+^ T cells recovered from brain tissues were similar (10^3^ to 10^4^/gram) regardless of HIV infection status, which was approximately 10–330 times less than that of MG from the same donor (Figure 2G). These data were consistent with the previous reports showing that MG are the major population of immune cells within the CNS (1).

**Figure 3:**
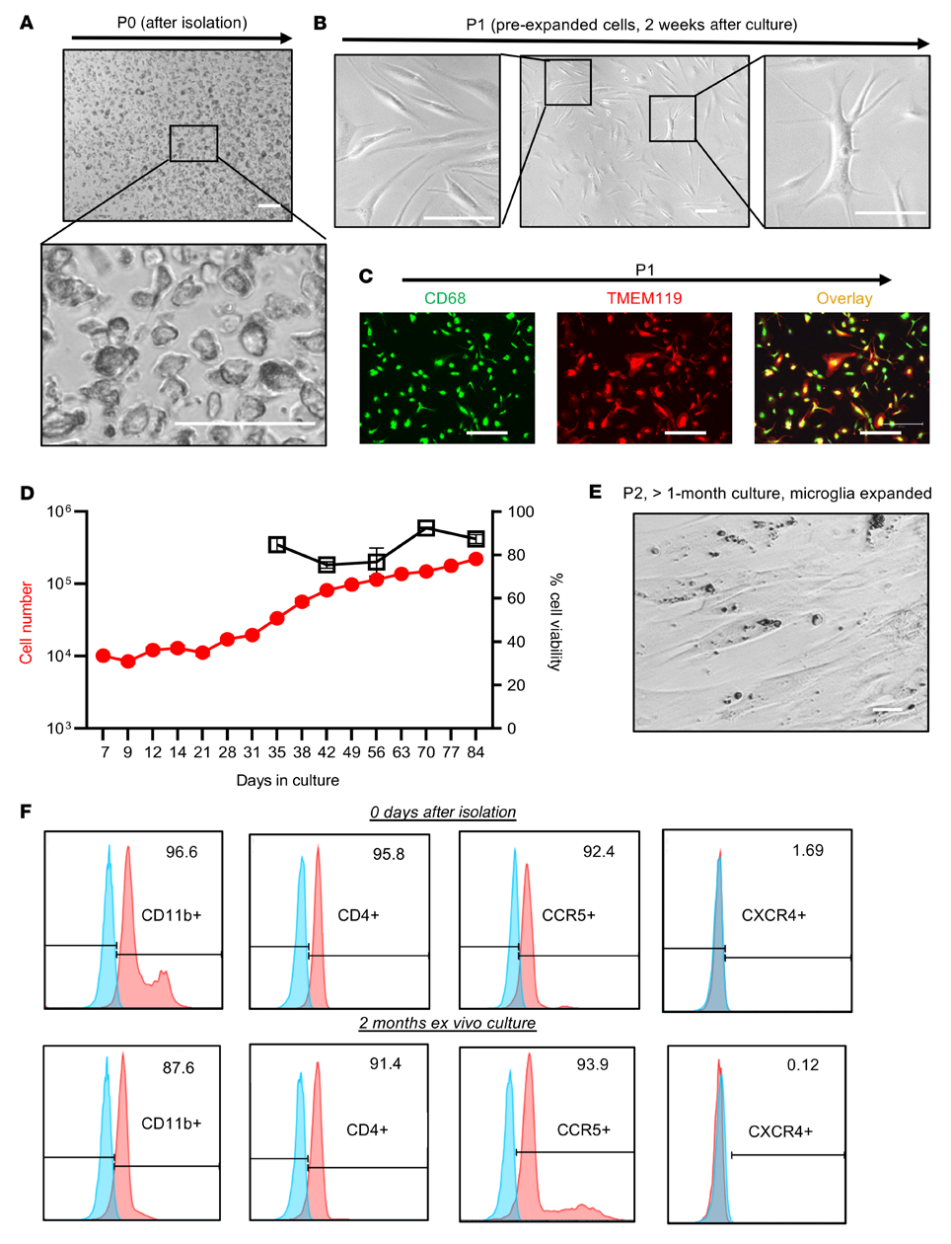
Characterization of MG from fresh postmortem brain tissues from PWH on ART. (**A**–**C**) Representative images show the morphology and/or phenotype of MG at the P0 (**A**) or P1 (**B**) stage during culture. The isolated P1 MG remaining expressed the BrMC marker CD68 (green) and the MG-specific marker TMEM119 (red), which largely overlapped with each other (yellow) (**C**). Scale bars: 100 μm (**A** and **B**) and 400 μm (**C**). (**D**) Human MG proliferated ex vivo (*n* = 3). (**E**) Image shows proliferation of P2 MG (after >1 month of ex vivo culturing). Scale bar: 100 μm. (**F**) MG at both P0 and P2 stages expressed the myeloid cell pan-marker CD11b, the HIV receptor CD4, and its coreceptor CCR5, whereas the CXCR4 coreceptor was undetectable. Unlabeled controls are shown in blue.

We next determined whether, as in NHPs, human cells isolated in this way could be maintained ex vivo in the presence of ART for sufficient periods of time to study the biology of persistent HIV infection. Figure 3A shows the morphology of MG immediately after isolation, termed phase 0 cells (P0), in which the purity of these isolated MG was greater than 95.5% CD11b^+^ (Figure 2, B and C) without T cell contamination (Figure 2E). During the 1–2 weeks of ex vivo culturing, the MG became firmly attached, forming a monolayer (Figure 3B). This short-term culture allowed the MG to initiate growth. The majority of these preproliferation cells (phase 1 [P1] cells) exhibited a small bipolar phenotype (Figure 3B, left panel), which persisted for up to 2 months in culture. Interestingly, a small portion of MG showed a more differentiated phenotype with multiple cell membrane extensions (Figure 3B, far right panel). Consistent with observations in MG isolated from NHPs and the characterization of human MG (Figure 1B and Figure 2C), more than 95% of the P1 brain MG cells remained TMEM119^+^ (Figure 2C and Figure 3C). While this pool of human MG proliferated at a low rate in the presence of ART, after 2 weeks of culturing, the cell numbers began to double every 1–3 weeks (Figure 3D). This growth rate was slower than that of MG derived from NHP brain (Figure 1D). We termed these human proliferating cells phase 2 (P2) cells (>2 weeks of ex vivo culture). As shown in Figure 3E, these proliferated MG still maintained a bipolar phenotype and could be trypsinized and passaged. The myeloid cell marker (CD11b) remained highly expressed in human-derived MG at both the P0 and P2 stages (Figure 3F). We were not able to measure CD11b in P1 cells by flow cytometry, as MG at this stage were adhered to the dish. MG phenotypes were maintained despite long-term ex vivo culturing. Isolated MG expressed the HIV receptor CD4 and its coreceptor CCR5, but expression of the alternate HIV coreceptor CXCR4 was not detected (Figure 3F), implying that MG may support the persistence of CCR5-tropic HIV.

Hence, the capacity to culture these human brain–derived MG ex vivo makes it feasible to rigorously address the key question of whether MG isolated from ART-suppressed PWH persistently harbor replication-competent HIV and thereby serve as a true, durable viral reservoir in the CNS. In the following studies, cell purity and proviral DNA content or HIV RNA expression were assayed using P0 cells, and latency reversal and replication-competent viral recovery were assessed in P1 cells. In this way, we were able to examine HIV persistence in MG, while minimizing ex vivo manipulation as much as possible.

### Quantification of cellular and integrated HIV DNA levels and detection of a transcriptionally active and transcriptionally latent HIV reservoir.

**Figure 4:**
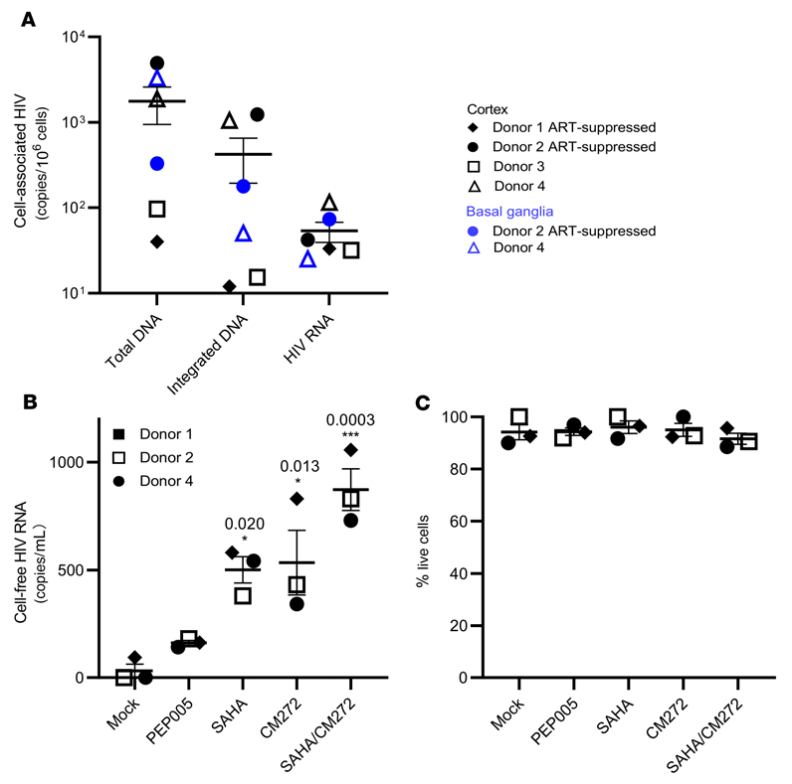
The frequency of HIV DNA and RNA in MG isolated from PWH and latency reversal after induction by epigenetic and nonepigenetic regulators. (**A**) Total HIV DNA, integrated HIV DNA, and cell-associated RNA were detectable in MG isolated from cortex and/or basal ganglia of HIV+ donors. Each symbol represents an average of 3 RT-qPCR measurements in MG (*n* = 4). (**B**) The response of human MG to LRAs. Cell-free HIV *gag* RNA in the cortical MG culture supernatant was measured on day 7 after LRA treatment. The HDACi SAHA (250 nM) and the methytransferase inhibitor CM272 (50 nM), alone or in combination, markedly induced HIV RNA release. *P < 0.05 and ***P < 0.001, by 1-way ANOVA (*n* = 3). (**C**) Cellular viability was measured by trypan blue exclusion (*n* = 3).

We initially measured persistent infection of isolated MG derived from PWH on ART by examining the levels of total HIV DNA by gag ddPCR and integrated proviral DNA by Alu-PCR. Total HIV DNA in isolated MG was detectable in MG isolates (P0) from all 4 donors, including the 2 fully ART-suppressed donors (donors 1 and 2, plasma viremia <30 copies on day 6 before death), with an average of 1,769 copies of HIV DNA/10^6^ MG, ranging from 40–4,950 copies/10^6^ MG. Compared with total HIV DNA, we observed a relatively low level of integrated HIV DNA in each of these MG populations, with an average level of 415 copies/10^6^ cells, ranging from 12–1,230 copies/10^6^ cells (Figure 4A), suggesting that most of the HIV DNA was present in the form of preintegrated HIV and/or 2 long-terminal repeat (LTR) circles.

The detection of both total HIV DNA and integrated proviral DNA is suggestive of HIV persistence in MG despite suppressive ART. To gain insight into the status of HIV in MG isolated from PWH on ART, we assessed cell-associated HIV RNA expression (37–39). We found that a much lower level of HIV RNA (gag) was detected in all MG samples, ranging from 25–115 copies/10^6^ MG (Figure 4A). At this point, it was not clear whether these transcripts were intact and replication competent or were the products of defective HIV genome expression. Notably, the levels of proviral DNA, integrated DNA, and viral RNA were similar between donors who were on ART until the time of death (donors 1 and 2) and donors with ART interruption (ATI) (donor 3, plasma viremia = 375 copies/mL) or without a record of plasma viremia (donor 4) close to the time of death (Figure 4A and Table 3). Given that the average levels of integrated proviral DNA (average of 422 copies/10^6^ MG) and total proviral DNA (average of 1,769 copies/10^6^ MG) were approximately 7.92- and 33.13-fold higher than viral RNA levels (average of 53 copies/10^6^ MG) (Figure 4A), the majority of microglial HIV appeared to be transcriptionally silent. This is similar to our observations in NHP MG (Figure 1F).

We have shown that epigenetic regulators such as the HDACi SAHA were able to reverse SIV latency (Figure 1F). We first confirmed that SAHA reversed latency in an established microglial HIV latency model (a gift from Brandon K. Harvey group at the NIH) (40), shown by LTR-driven HIV expression (Supplemental Figure 3). We then cultured human MG for 2 weeks ex vivo in the presence of ART to allow the cells to recover and initiate the growth process. After the cessation of ART, the cells were treated with latency reversal agents (LRAs) for 7 days. As expected, SAHA consistently induced cell-free viral RNA production in MG isolated from PWH (donors 1, 2, and 4) compared with DMSO treatment. The NF-κB agonist PEP005 (34) was less effective in the induction of viral RNA expression. The methylation inhibitor CM272 (41) also induced HIV RNA expression and augmented SAHA-induced latency reversal by 1.74-fold (Figure 4, B and C). Thus, latent HIV infection in MG appeared to differ from that in T cells, with an increased response to epigenetic regulators, including protein deacetylation and methylation.

### Inducible viral RNA outgrowth in MG but not CNS T cells from PWH on ART.

**Figure 5:**
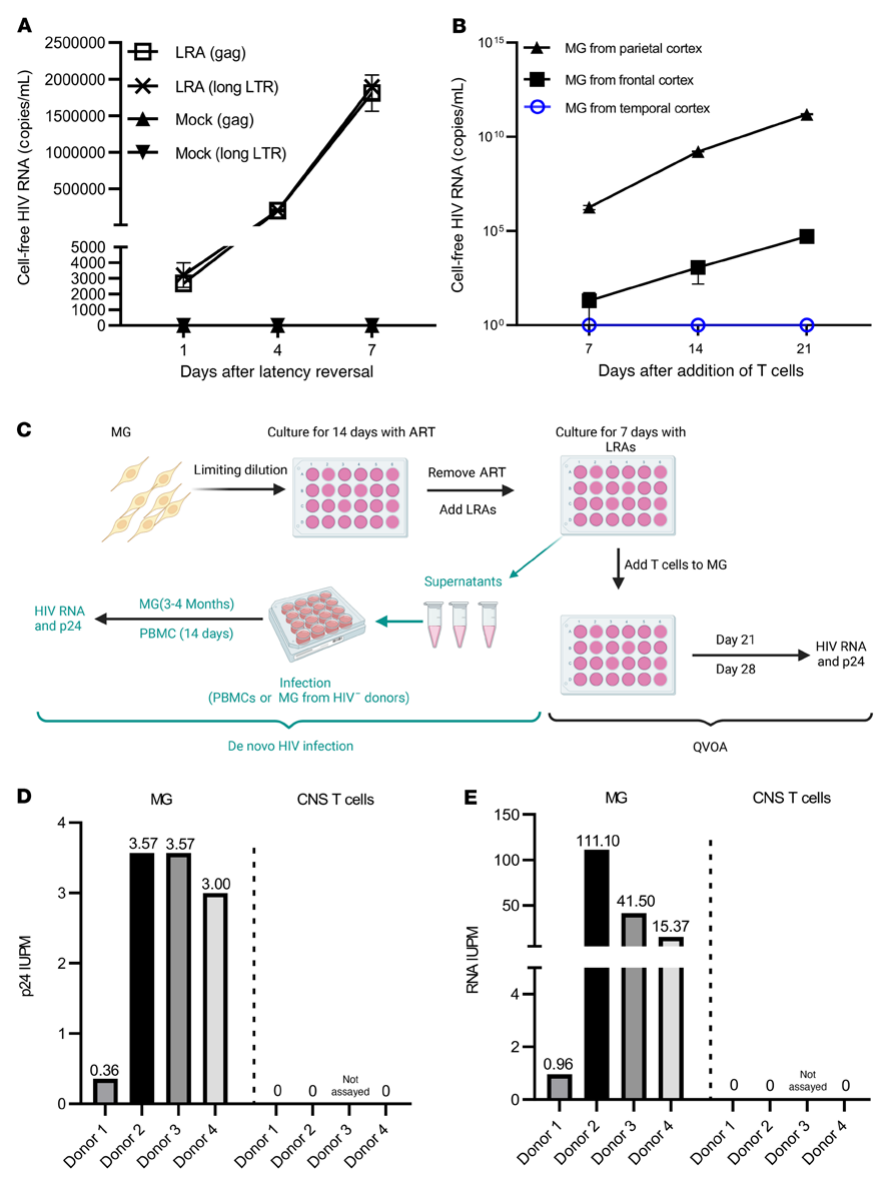
HIV was outgrown from brain-derived MG. (**A**) Cell-free HIV RNA (*gag*) in the culture supernatants was measured in the SAHA- and CM272-treated MG cultures (10^5^ cells/well) isolated from the parietal cortex of donor 2 (*n* = 3). (**B**) Outgrowth HIV was tracked over time by measuring viral RNA release in the culture supernatant of SAHA- and CM272-treated MG wells (from the indicated brain region of donor 2) after addition of CD8-depleted PHA PBMC blasts (*n* = 3). (**C**) MG QVOA and de novo infection by human brain MG–derived HIV. After MG were isolated from the brain of PWH, cells were plated in the 24-well plates with limited dilutions and cultured for 14 days in the presence of ART, allowing the cells to settle down and attach to the surface. The latent HIV was activated with SAHA and CM272 for another 7 days, and then the LRAs were washed out. For the MG QVOA, LRA-treated MG were cultured with CD8-depleted, HIV^–^ PBMC PHA blasts or MOLT-4/CCR5 cells. Viral outgrowth was measured on day 21 and was further confirmed on day 28. De novo HIV infection was used to assess MG-derived, replication-competent HIV via inoculation of virus from LRA-stimulated MG culture into MG or PBMC blasts isolated from HIV^–^ donors. HIV replication was assayed by HIV RNA and p24 released into culture supernatants. ART consisted of raltegravir plus darunavir plus nevirapine. (**D** and **E**) The IUPM of MG and CNS T cells was calculated by standard viral outgrowth assay (measuring HIV p24 antigen release in the wells) (**D**) and by RT-ddPCR to measure viral RNA^+^ wells (**E**) (*n* = 4).

We performed an inducible viral outgrowth assay to assess the replication-competent reservoir in human MG. For the most rigorous analysis, we studied MG isolated from donor 2, who remained virally suppressed until death (Table 3). Human MG at P0 were then plated at 10^5^ cells per well and cultured for 14 days in the presence of ART, enabling MG to attach to the culture plate and initiate the growth process as shown in Figure 3B. We then removed ART and stimulated the cells with the combination of SAHA and CM272, given our observations suggesting that this pair of epigenetic modulators might maximally induce latency reversal (Figure 4B). MG isolated from the parietal cortex of donor 2 (Figure 5A) yielded cell-free viral RNA as early as 1 day after treatment, and we detected an exponential increase in cell-free viral RNA release on days 4 and 7. To further propagate outgrowth viruses, we next added CD8-depleted PBMC phytohemagglutinin (PHA) blasts isolated from a donor without HIV to the LRA-treated MG isolated from different brain regions. As before, exponential viral RNA outgrowth (>10^10^ copies/mL) was observed in MG from the parietal cortex for 21 days (Figure 5B). In addition, low levels of viral outgrowth were detected in MG isolated from the frontal cortex but was not detectable in MG from the temporal cortex in this donor (Figure 5B).

Given that cell-free HIV RNA outgrowth from MG was detectable, we sought to quantitate the frequency of this inducible HIV reservoir. As outlined in Figure 5C, human MG from both PWH on ART (donors 1 and 2) and off ART (donors 3 and 4) were plated in limiting dilution and cultured for 14 days in the presence of ART to allow cell expansion but not viral spread. As the combination of SAHA and CM272 induced maximal latency reversal in MG (Figure 4B), ART was removed, and cells were treated with this combination for 7 days. CD8-depleted PHA blasts from a donor without HIV (42) or HIV-permissive cells (e.g., Molt4/CCR5) (43) were then added to cultures passaged for up to 28 days. We performed a p24 antigen assay to measure the reservoir frequency, reported as infected units per million (IUPM) in MG (Figure 5, C and D, and Supplemental Table 3). Using the maximum likelihood method, the IUPM for ART-suppressed donors 1 and 2 was 0.36 and 3.57 (Figure 5D and Supplemental Tables 3 and 4), respectively, within the range of what is observed in T cells (42, 44, 45). Interestingly, during coculturing of MG with CD8-depleted PBMCs, MG did not proliferate as well as when MG were cultured alone. Also, the value of IUPM was calculated on the basis of original P0 cells plated in the wells. Therefore, IUPM should not be overestimated, even though MG were cultured for 14 days and could proliferate prior to LRA treatment. Conversely, as p24 assays may underrepresent the potential release of infection virus, for added rigor, we estimated the reservoir of viral RNA in the same cultures. Maximum likelihood calculations using HIV RNA^+^ wells (Figure 5E and Supplemental Tables 3 and 4) yielded a possible reservoir IUPM frequency in MG as high as 3.57–111.10 for donor 2 and 0.36–0.96 for donor 1. We also measured the HIV reservoir in MG isolated from donors 3 and 4, although it should be taken into consideration that ART was interrupted in these donors shortly prior to death (Table 3). Although viral RNA was low in the isolated P0 MG (Figure 5A), we detected outgrowth in isolated MG, with p24 IUPMs of 3.57 and 3.00 in donors 3 and 4, and IUPMs of 41.50 and 15.37, respectively, were estimated using viral RNA values (Figure 5, D and E, and Supplemental Tables 5 and 6).

Notably, although T cells have been identified as latent and persistent viral reservoirs, which has been extensively studied in peripheral blood, we found that CD3^+^ T cells were rare in the brain tissues. In donor 2, sequential isolation procedures (Figure 1A) yielded only 23,500 CD3^+^ T cells from 33.68 grams of brain tissue. In contrast, 7.13 million viable MG were isolated from the same tissue. On average, 1 gram of brain tissue yielded 6,739 CD3^+^ T cells and 281,533 CD11b^+^ MG. For comparison, we conducted a viral outgrowth assay (33) using T cells isolated from CNS tissues from donor 2. However, no viral outgrowth was detected using all available 23,500 CNS T cells (Figure 5, D and E), despite the fact that viral outgrowth was detected in CD4^+^ T cells isolated from 20 million PBMCs from the same donor. The lack of viral outgrowth from brain T cells was also found in donor 1 (1.4 × 10^5^ CNS T cells from 65 grams of brain tissue) and donor 4 (1.06 × 10^6^ CNS T cells from 52 grams of brain tissue; Figure 5, D and E). Taken together, given the limited number of available CNS T cells, it is not at all surprising that no HIV was recovered, as we were able to assay 40 times more peripheral T cells than CNS T cells. However, these observations suggest that MG may constitute the major HIV reservoir within the CNS in this cohort study.

### MG-derived HIV was replication competent in both myeloid cells and CD8-depleted PBMCs.

**Figure 6:**
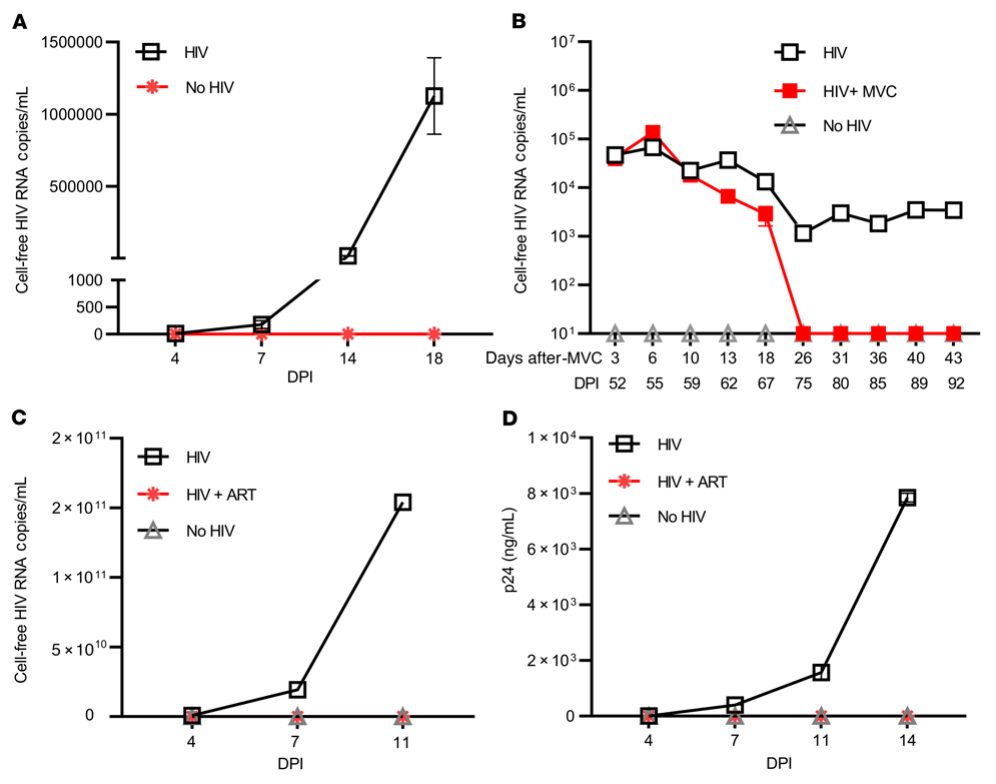
Outgrowth HIV reestablished its infection in both myeloid cells and PBMC PHA blasts. Supernatant HIV from day 7 after CM272- and SAHA-stimulated reinfected MG culture (as shown in Figure 4A) (**A** and **B**) or CD8-depleted PBMC PHA blasts isolated from HIV^–^ donors (**C** and **D**). The same sources of target MG cultures or PHA blasts without addition of HIV (No HIV) were used as negative infection controls. HIV production was measured by HIV RT-ddPCR (**A**–**C**) or HIV p24 ELISA (**D**). HIV infection in MG was suppressed by the CCR5 inhibitor MVC (**B**), while ART (raltegravir/darunavir/nevirapine) treatment blocked HIV infection in PBMC PHA blasts (**C** and **D**) (*n* = 3). DPI, days post infection.

Given the detection of replication-competent HIV in brain MG, we sought to characterize the properties of viral isolates collected from MG (from donor 2) supernatants on day 7 after LRA treatment. We tested whether this isolate could infect MG isolated from the brain of an HIV^–^ donor enrolled in the NDRI. We observed exponential viral replication in MG infected with HIV, but not in MG that were mock infected (Figure 6A). We consistently detected persistent viral release in MG culture for over 3 months. Notably, HIV infection in MG was completely suppressed by the CCR5 inhibitor maraviroc (MVC) (46) (Figure 6B). These data demonstrate that MG-derived HIV productively infects MG, is released for long periods of time, and uses the CCR5 HIV coreceptor.

The same LRA-induced HIV from MG was also used to infect HIV^–^, CD8-depleted, PHA-treated PBMCs with the same MOI. We observed exponential viral replication by measurement of both HIV RNA (Figure 6C) and p24 release in culture supernatant (Figure 6D). In fact, the level of viral RNA from infected PBMCs (Figure 6C) was 4–5 logs higher than in MG cells (Figure 6A), suggesting that these MG-derived viruses can more efficiently replicate in T cells compared with the infection in MG. Of note, HIV infection of PBMC blasts was effectively blocked by ART (Figure 6, C and D). As latent HIV released by brain MG from PWH on ART can infect both myeloid cells and T cells, virus in the MG reservoir has the capacity to serve as the source of rebound viremia after ARTi and may spread rapidly in T cells.

### Viral sequence analyses suggest a CNS origin of MG outgrowth virus.

**Figure 7:**
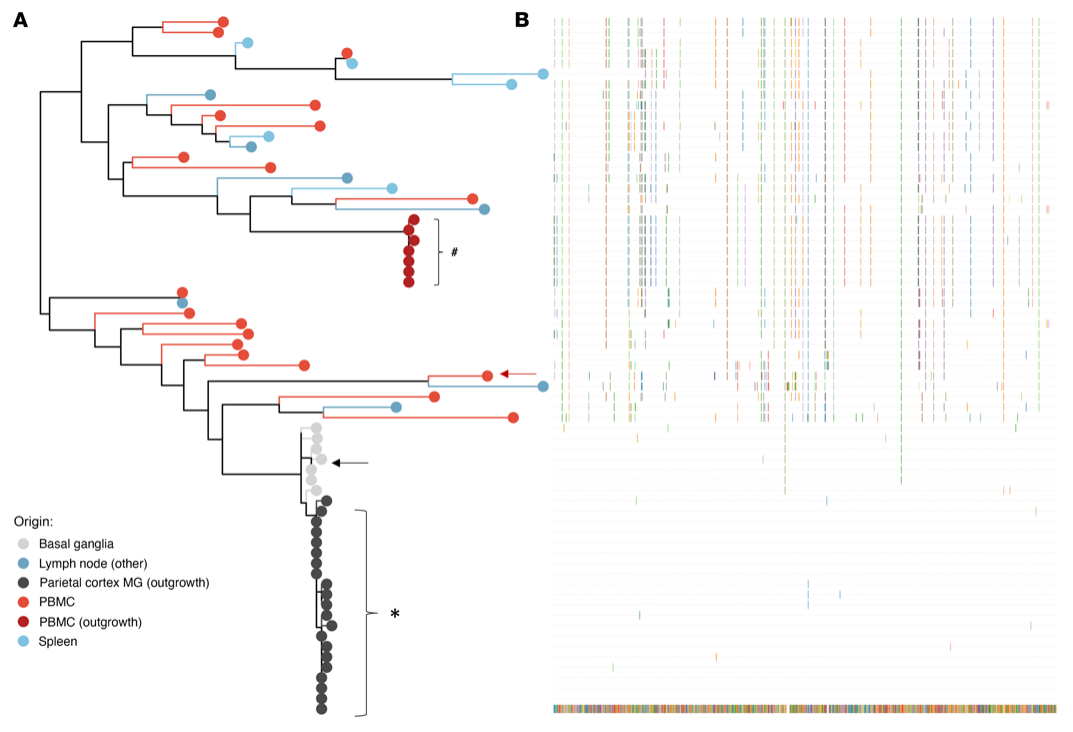
Viral sequence analysis of MG outgrowth HIV from donor 2. (**A**) Maximum likelihood phylogeny of the intact FL envelope sequences. Maximum likelihood phylogeny reconstruction was performed by IQtree (77). Tree topology confirmed that the MG outgrowth viral population (isolated from parietal cortex, in black) was more closely related to viral sequences from brain tissues (basal ganglia, gray) but distinctly related to viral sequences in the PBMCs (red) as well as lymphoid organs (spleen and lymph nodes, blue). The plot was generated with ggtree R package (78). Arrows indicate the *envs* that were cloned from proviral DNA and examined in the phenotyping assays. Additional clones were generated from the MG and PBMC outgrowth cultures. The clone from the MG outgrowth culture was identical to the MG OGV sequences, and the clone from the PBMC outgrowth culture was identical to 4 PBMC OGV sequences. ^#^Sequences from OGV. (**B**) Viral sequences were aligned to MG-OGV.

To further characterize the genotype and phenotype of HIV recovered from MG of ART-fully-suppressed donor 2, we next analyzed viral envelope sequences after single-genome amplification (SGA) of outgrowth virus (OGV) from MG and PBMCs and DNA from brain tissues, PBMCs, and peripheral tissues from the same donor (Figure 7A and Table 4). As expected, the HIV-intact full-length (FL) envelope sequences from the MG outgrowth culture were nearly identical. Similarly, the seven envelope sequences from the PBMC outgrowth culture were nearly identical to each other. Proviral DNA sequencing was used to generate 7 intact FL envelope sequences from basal ganglia tissue, 19 from PBMCs, 7 from lymph nodes, and 6 from spleen (Table 4). Sequences of MG OGV (isolated from the parietal cortex) were more closely related to proviral sequences in basal ganglia tissues than to viral sequences in PBMCs or lymphoid organs (Figure 7, A and B), consistent with the seeding of HIV in the CNS. The HIV DNA diversity was significantly lower in CNS tissues and MG outgrowth HIV compared with PBMC sequences and with the sequences obtained from spleen and lymph nodes (Table 4). This was confirmed by tree topology (Figure 7A) and sequence alignment (Figure 7B).

**Figure 8:**
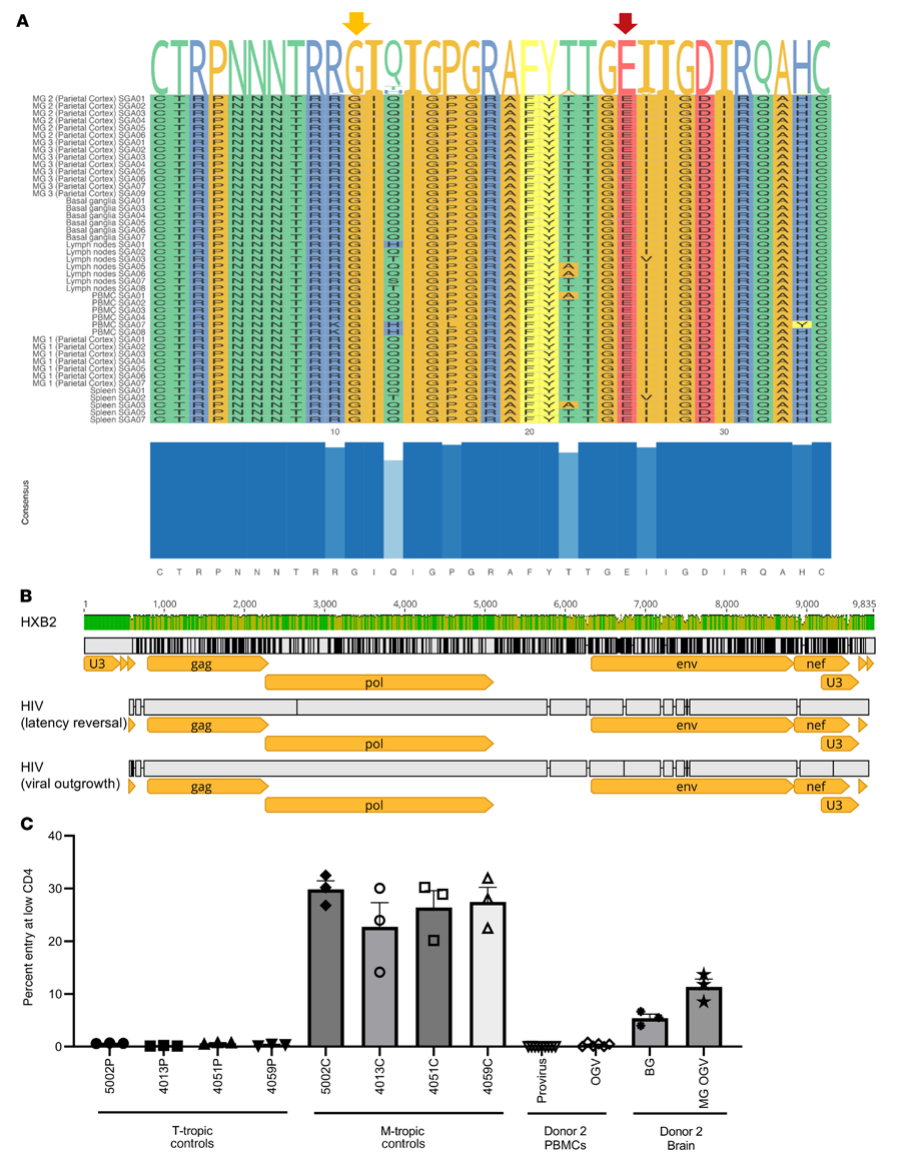
Phenotyping of brain MG outgrowth HIV from donor 2. (**A**) Amino acid variations of the V3 regions from parietal cortex MG, basal ganglia, and PBMCs. The amino acid positions 11 and 25 (arrows) were conserved in all sequences predicted to be CCR5-tropic using geno2pheno (47), a conservative 10% false-positive rate threshold for coreceptor CXCR4 usage based on the recommendation from the European Consensus Group on clinical management of HIV tropism testing. The plot was created by the ggmsa R package (79). Amino acid variations are presented at the top of the sequence alignment, and the V3 consensus is depicted at the bottom. Only 4 positions (10, 13, 22, and 34) differed across all V3 sequences. (**B**) Near-FL HIV genomes were recovered from MG derived from donor 2 after latency reversal (day 7), and supernatants of PBMCs infected with the MG-induced HIV (day 14). (**C**) HIV tropism was determined by the ability of luciferase reporter viruses to enter Affinofile cells expressing a low density of CD4 relative to their ability to enter Affinofile cells expressing high levels of CD4 (2, 49, 51). Reporter pseudoviruses were generated using *envs* cloned from MG and PBMC outgrowth cultures and proviral DNA in PBMCs and basal ganglia tissue, all from donor 2 (cloned *envs* shown in Figure 7A). Entry phenotypes were then compared with well-characterized T- and M-tropic controls cloned directly from patient samples (2, 49).

We also interrogated the coreceptor usage of HIV sequences by applying the Geno2Pheno algorithm (47). All variants were predicted to be CCR5 tropic (Figure 8A and Table 4). This predication was consistent with the earlier observations that MG expressed the CCR5 HIV coreceptor (Figure 3F) and that the infection of HIV in MG was completely blocked by the CCR5 inhibitor MVC (Figure 6B).

We also obtained a near-FL sequence of MG OGV HIV from donor 2 through amplification by nested PCR of overlapping 5′ and 3′ half-genomes (48, 49) (GenBank accession nos. OQ325479 for HIV induced from MG and OQ325480 for HIV from MG OGV). As shown in Figure 8B, we sequenced both MG-induced virus (MG virus from 7 days after LRA exposure, as shown in Figure 4A) and PBMC-expanded OGV (14 days after expanded LRA-induced virus in CD8-depleted PBMCs, as shown in Figure 5B). The sequences obtained from these 2 cultures were almost identical, indicating that ex vivo culturing induced few mutations into the HIV genome and that OGV truly reflected the viral sequences present in vivo.

### MG-derived HIV represents a CNS-specific lineage that has adapted to replication in myeloid cells.

Macrophages express a lower surface density of CD4 than do CD4^+^ T cells (50), explaining why viruses adapted to replication in macrophages (i.e., M-tropic viruses) have an enhanced ability to enter cells expressing low densities of CD4, whereas viruses adapted to replicate in CD4^+^ T cells (i.e. T-tropic viruses) are inefficient at entering cells with low CD4 receptor density (50, 51). The ability to infect Affinofile cells expressing a low density of CD4 is a proxy for M-tropism (50, 52). Like well-characterized M-tropic *envs* cloned from the CNS of humans (2, 50, 51), an *env* cloned from the outgrowth culture of MG and an *env* cloned from a provirus in the basal ganglia facilitated efficient entry of cells expressing a low density of CD4 (Figure 8C)_._ In contrast, 2 *envs* cloned from the PBMC outgrowth and a PBMC-derived provirus from the same donor were unable to efficiently enter cells expressing low levels of CD4. The fact that both clones derived from the CNS were genetically and phenotypically distinct from variants in the periphery provides strong evidence that HIV represents a CNS-specific lineage that has adapted to replication in MG.

## Discussion

Whether CNS cells, particularly brain MG, can durably harbor replication-competent HIV in PWH on long-term, fully suppressive ART and thereby serve as true viral reservoirs has not been clearly demonstrated. Direct measurement of replication-competent HIV in the brain MG requires a pure population of viable cells derived from CNS tissue from PWH on ART. In addition to the obvious ethical obstacles regarding the sampling of human brain tissues, there are technical hurdles in the isolation of highly pure MG. Isolation and culturing of BrMCs and MG have been widely used in rodent models (53–59); however, these methods have been less frequently conducted with brains from rhesus macaques or humans. In this study, we adopted methods from previous studies involving rodent models (53–59) and rhesus macaques (12, 15) and established protocols to isolate brain MG and characterize viral reservoirs in the NHP model of infection, which we then optimized and applied to human brain tissues donated to the Last Gift program. This unique cohort has recruited altruistic PWH with terminal illness, who wished to participate in HIV cure research at the end of life, including tissue donation for a rapid research autopsy within 6 hours of death (60–62). We also accessed brain tissue (principally from people without HIV) through the NDRI. With these brain tissues from both PWH and people without HIV, we achieved successful isolation and ex vivo culturing of MG, principally TEME119^+^ MG. In addition, we observed that brain-derived MG may proliferate ex vivo, which may enhance the ability to study these cells. We were thus able to examine the role of MG as stable reservoirs of persistent HIV infection in the CNS.

To ensure that replication-competent HIV was truly derived from brain MG, we first used highly sensitive RT-qPCR and ddPCR assays with the detection limit of 1 T cell per million MG. Our data showed that RNA for the T cell marker CD3 was undetectable in isolated MG. Of note, in our virological studies, we assessed fewer than 1 million MG per culture, ensuring that there was likely to be less than 1 T cell in our purified MG during our reservoir assay. Second, we performed sequential selection for T cells followed by MG selection and subsequent side-to-side viral outgrowth assays with these isolated CNS cells at 2 separate tissue culture facilities. Our results revealed that, in samples from donors 1, 2 and 4, viral outgrowth was not detected in the pooled CNS T cells. In contrast, replication-competent HIV was recovered from the isolated MG. Third, we found that CNS T cells were rare compared with MG in samples from the same donors (<10^6^ CNS T cells from 20–30 grams of brain tissue, in which 10–1,000 times more MG were isolated from the same tissue sources). Although the CNS T cell counts were low in brain tissues from donors, this may have been independent from the immunosuppression status, since CNS T cell numbers in brain tissues were comparable between PWH on ART and HIV-uninfected donors.

It has been reported that T cells in the brain may provide an HIV reservoir in an HIV-infected Hu mouse model of HIV latency (6). Unfortunately, we were unable to isolate a sufficient number of T cells (<1 million total T cells/sample) from brain tissues from each person with HIV to determine whether they contained HIV. Since CD3^+^ CNS T cells were used for quantitative viral outgrowth assay (QVOA), the presence of CD8^+^ T cells within the isolated CNS T cells might impair viral outgrowth. Nevertheless, although CNS-resident CD4^+^ T cells may harbor latent HIV, the frequency of recovery of HIV from MG suggests that MG are a substantial reservoir of persistent, replication-competent HIV in the brain. In addition, sequence analysis of MG OGV showed that it was most closely related to viral sequences identified in the brain tissues of the same donor. Finally, variants from MG outgrowth culture and proviral DNA in the basal ganglia had adapted to infecting brain myeloid cells (i.e., were M-tropic), a phenotype typically observed in cerebrospinal fluid–derived HIV (63) and distinct from a T-tropic, PBMC-derived variant from the same participant. These rigorous analyses indicate that MG from a donor on ART harbored replication-competent HIV belonging to a CNS-specific viral lineage that had adapted to replication in MG and macrophages.

Our analysis revealed that during suppressive ART, the basal levels of cell-associated viral RNA were very low in brain MG isolated either from NHPs or humans. Importantly, SIV RNA was up to 6-fold lower than SIV DNA levels in NHP brain MG, while HIV RNA was up to 33-fold lower than HIV DNA levels in human MG. These observations suggest that proviral HIV in the brain MG is largely silent in PWH on ART. Nevertheless, we found that, as in peripheral cells, HIV latency in the brain MG could be disrupted by epigenetic modulators such as HDACi and a methylation inhibitor. This is consistent with results in model systems in which epigenetic factors such as the corepressor COUP-TF–interacting protein 2 (CTIP2) (64) and CCAAT/enhancer-binding protein β (C/EBP-β) (65) can suppress HIV or SIV transcription in brain microglial systems by downmodulating histone acetylation and upregulating histone methylation at the HIV LTR. It has been shown that CTIP2 regulates HIV latency by interacting with HDACs to promote histone deacetylation (64, 66) and that C/EBP-β suppresses histone acetylation at the SIV LTR, leading to viral latency in the brain of SIV-infected macaques (65, 67). Increased levels of CTIP2, HP1, MeCP2, and HDAC1 were observed in the human brain (66). Most recently, Nurr1 was shown to silence MG HIV after its recruitment of the co-repressor of repressor element 1–silencing transcription (CoREST) repressor complexes HDAC1-G9a-EZH2 to the HIV LTR (68). Consistent with these observations, our data suggest that histone deacetylation and methylation may contribute to proviral quiescence and latency within the MG population in the human brain. Conversely, while NF-κB signaling has been identified as one of the most important HIV transcription pathways to induce HIV expression from latency in CD4^+^ T cells ex vivo and in vivo (69, 70), we were surprised to find that NF-κB activators were less effective than epigenetic regulators in the induction of HIV in MG, which was consistent with the low levels of NF-κB observed in such cells (71–73).

HIV recovered from MG could directly infect both MG and CD8-depleted PBMCs, although HIV replication in PBMCs remained 4–5 logs higher than in MG, pointing to intrinsic differences in viral replication in these cell types. MG isolated from HIV^–^ donors supported HIV replication for more than 4 months in vitro (our unpublished observations). HIV active and latent infection in human MG characterized in this study could serve as useful tools for the rigorous examination of infection, latency, and other mechanisms of viral biology in MG models in vitro. Since the circulating T cells are highly permissive to MG-derived HIV, T cells could be the main source to drive viral rebound after ARTi as previously described (18).

We were able to recover near-FL HIV from MG from 1 donor on ART, illustrating that brain MG can serve as persistent, inducible reservoirs of HIV. Our observation that a variant cultured from MG in the parietal cortex was genetically similar to HIV proviruses in another region of the brain (basal ganglia) suggests that these reservoirs were established at approximately the same time and did not diverge substantially. This could happen if virus produced in 1 brain region infects cells in other regions and then stops replicating as a result of the initiation of ART and/or the establishment of latency. Regardless, our results indicate that a macrophage-tropic lineage can seed reservoirs across the brain.

Although this study is limited by the small number of available samples from human donors on ART, we believe the findings in this rare and unique cohort are notable and consistent with NHP studies. Our observations support the concept that brain MG are a stable reservoir of quiescent infection and may be a source of viral rebound upon treatment interruption. Future efforts to clear HIV infection will have to include assessments of the persistence of HIV within CNS MG.

## Methods

### Isolation and culturing of BrMCs and MG

Isolation and culturing of BrMCs have been widely used for studies in rodent models (53–59). Recently, these techniques were used in the rhesus NHP model in a few studies (12, 15, 74, 75). On the basis of these published protocols, we established a technique that included enzymatic and mechanical steps to dissociate brain tissues and a myelin debris removal step to generate CNS single-cell suspensions. Each step was optimized carefully to achieve the high yield of viable cells with low cell damage.

The brain tissue pieces were rinsed with 1× HBSS, and meninges and blood vessels were removed. The tissue pieces were diced into 0.2 cm^3^ pieces and treated with 0.25% trypsin-EDTA with 10 μg/mL DNase I in 1× HBSS and incubated at 37°C for 1 hour with agitation. After incubation, the enzymes were inactivated by adding 10% FBS (volume/volume). The digested tissue pieces were triturated with a 10 mL pipette 10 times followed by a 5 mL pipette 10 times. To ensure a single-cell suspension, the dissociated tissue solution was passed through a spinal needle using a 20 mL syringe. After the tissue dissociation steps, the myelin debris layer was removed by 35% Percoll density gradient as described before (76). The single-cell suspension of CNS cells was then generated. The cells were resuspended in 5 mL ACK lysis buffer for 10 minutes to further eliminate RBCs. The cells were counted on a Countess II (Invitrogen, Thermo Fisher Scientific), and then the viable cells were determined by a trypan blue exclusion assay. An aliquot of prepurified CNS cells was used for flow cytometric measurement as described below.

The T cells were then depleted from the isolated CNS cells by CD3^+^ selection. For human samples, the EasySep Release Human CD3 Positive Selection Kit (catalog 17751, STEMCELL Technologies) was used. For NHP samples, the CD3 MicroBead Kit, nonhuman primate (catalog 130-092-012, Miltenyi Biotec) was used. The selected CD3^+^ T cells were used to conduct comparative studies of the CNS reservoir in T cells.

Finally, BrMCs were purified from the CD3^+^ T cell–depleted CNS cell population. For human samples, CD11b MicroBeads, human and mouse (catalog 130-049-601, Miltenyi Biotec) were used. For NHP samples, CD11b MicroBeads, NHP (catalog 130-091-100, Miltenyi Biotec) were used (75). For NHP samples, further sequential selection was performed for MG by selecting TMEM119^+^ cells. An aliquot of T cell–depleted CNS cells or CD11b^+^ BrMCs (0.5–5 million) was rinsed with 1× HBSS. The cells were than incubated with Fc blocker (BioLegend) for 15 minutes at room temperature, and the blocker was then removed by washing with FACS buffer. The cells were incubated with anti-TMEM119 (extracellular) antibody (catalog 853301, BioLegend) for 15 minutes at room temperature, followed by incubation with an FITC-conjugated secondary antibody (catalog 115-097-003, Jackson ImmunoResearch) for another 15 minutes. The TMEM119^+^ cells were then selected with an FITC^+^ selection kit II (STEMCELL Technologies). For human samples, TMEM119 immune purification for MG was not performed because of the low yield and limited amount of available brain tissues.

To prepare BrMC cultures for further measurement of viral outgrowth and the responses to LRAs, the isolated BrMCs and MG were cultured in DMEM/F12 medium supplemented with 10% FBS and 1% l-glutamine, sodium pyruvate, HEPES buffer, GlutaMax, antibiotic-antimycotic, and 10 ng/mL M-CSF (PeproTech). For the cells from PWH, ART (200 nM raltegravir, 100 nM nevirapine and 25 nM darunavir from the NIH) was included in the culture.

### Statistics

All statistical analysis were performed using GraphPad Prism, version 9.3 (GraphPad Software). Where appropriate, results are expressed as the mean ± SEM. An unpaired or paired, 2-tailed Student’s *t* test or 1-way ANOVA multiple-group comparison was used to compare differences between groups. A *P* value of less than 0.05 was considered significant in all analyses performed.

### Study approval

#### Animal cohort.

Rhesus macaques (*Macaca mulatta*) were obtained from the Emory National Primate Research Center (ENPRC). Four animals were intravenously infected with 1 × 10^5^ TCID_50_ SIV_mac239_. Eight weeks after infection, ART (consisting of 2 reverse transcriptase inhibitors, tenofovir disoproxil fumarate and emtricitabine, plus the integrase inhibitor dolutegravir) was initiated to suppress the replication of SIV. ART was maintained until the time of necropsy, when brain tissues were collected. The animals reached undetectable viremia (<60 copies/mL) between weeks 10 and 32 of ART. For an 11- to 12-week period starting 81–87 weeks after infection, these animals also received a LRA and SIV-specific monoclonal antibodies in the presence of ART. Undetectable viral loads were confirmed at the time of necropsy 101–105 weeks after infection. Animals’ brain tissues from different regions were collected after necropsy and shipped to the UNC HIV Cure Center via overnight delivery. CNS cells were then immediately isolated from these tissues. The rhesus macaques were housed at the ENPRC and treated according to Emory University and ENPRC IACUC regulations (PROTO201700286). The animal care facilities are accredited by the US Department of Agriculture and the Association for Assessment and Accreditation of Laboratory Animal Care International (AAALAC). The care and use of animals were approved by the IACUC of Emory University.

#### Last Gift cohort.

Four participants with HIV and 4 participants without HIV were enrolled in this study. Three PWH (donors 1, 2, and 3) were from the Last Gift cohort. One additional individual with HIV (donor 4) as well as 4 donors without HIV were recruited through the NDRI. The Last Gift program enrolled altruistic, terminally ill PWH on ART for close perimortem follow-up. The inclusion criteria for the individuals in this study were: diagnosis of HIV with less than 6 months to live; on suppressive ART; and no CNS malignancy or immune checkpoint chemotherapy. The study was approved by the IRBs of the UCSD (IRB no. 160563) and the NDRI.

## Author contributions

GJ and YT conceived the study. GJ, YT, and DMM designed the experiments. SG, A Chaillon, DMS, MP, CI, BW, and JJE collected samples. YT, A Chaillon, LMW, DL, TLS, DZ, JD, EDLPP, JK, BA, MLC, MM, ALS, GDW, VS, AD, KJB, A Chahroudi, SBJ, and NMA performed the experiments. YT, GJ, DMM, A Chaillon, SG, KJB, A Chahroudi, SBJ, and NMA analyzed the data. YT and GJ wrote the manuscript. A Chaillon, SG, KJB, A Chahroudi, SBJ, NMA, JJE, DMM, YT, and GJ edited the manuscript.

## Supplementary Material

Supplemental data

## Figures and Tables

**Table 1:**
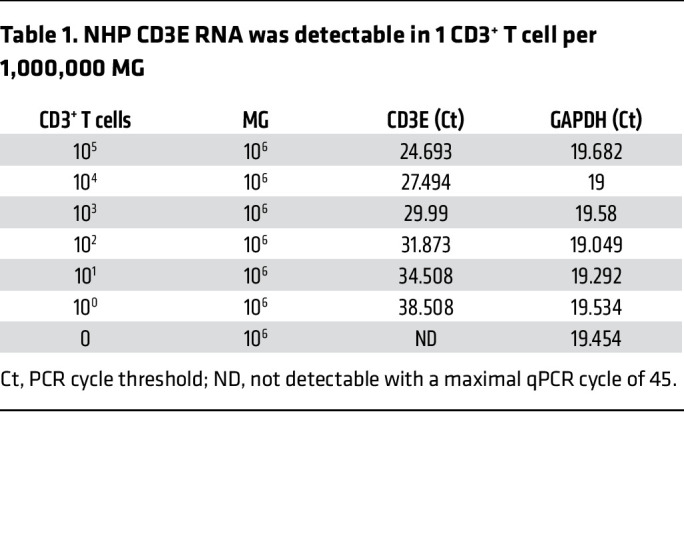
NHP CD3E RNA was detectable in 1 CD3^+^ T cell per 1,000,000 MG

**Table 2:**
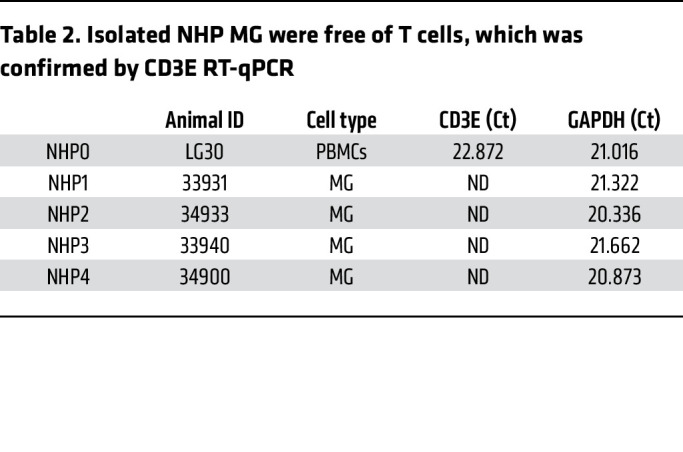
Isolated NHP MG were free of T cells, which was confirmed by CD3E RT-qPCR

**Table 3:**
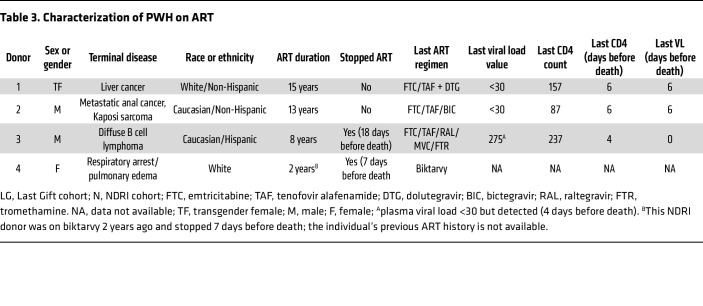
Characterization of PWH on ART

**Table 4:**
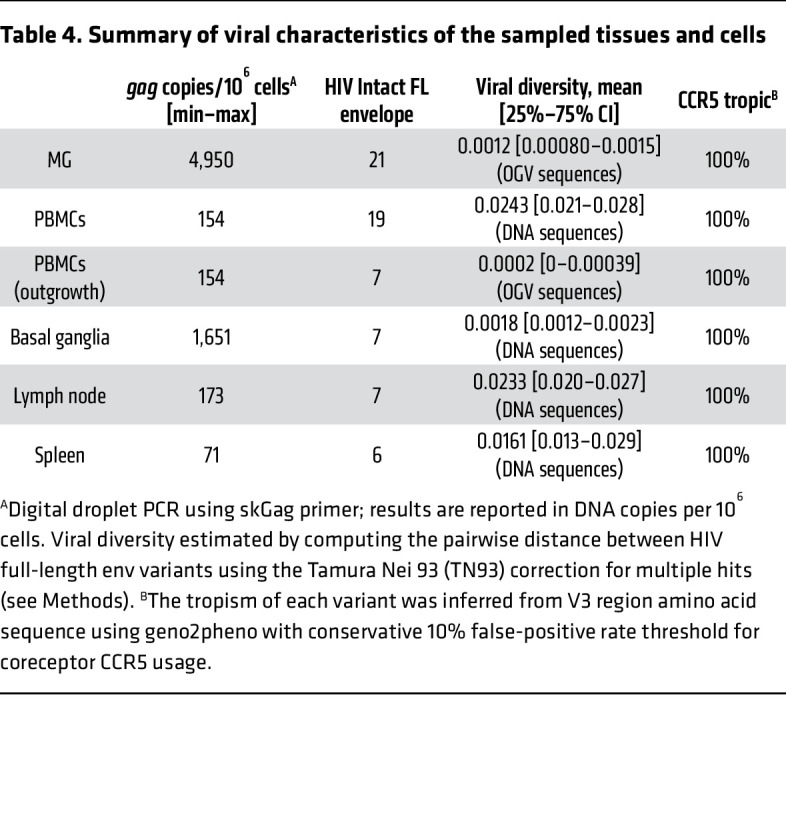
Summary of viral characteristics of the sampled tissues and cells

## Version 1 06/15/2023

Electronic publication
