# Supplementary material for: Brain microglia serve as a persistent HIV reservoir despite durable antiretroviral therapy
Source: J Clin Invest. 2023 Jun 15;133(12):e167417. doi: 10.1172/JCI167417 (PMC10266791; doi:10.1172/JCI167417)
Supplement: Supplemental data [file jci-133-167417-s043.pdf]

Supplementary Materials

Collection of fresh brain tissues

The brain tissues were collected from the donors within 6 hours of death for the individuals from the "Last Gift" cohort (Donors 1, 2, and 3) and less than 24 hours after death for the donors from NDRI (Donor 4 and four donors without HIV). The brain tissues from ART-suppressed SIV-infected rhesus macaques were collected immediately at necropsy. We obtained up to 20 gram tissue pieces from each brain region. Tissue pieces were collected in a sterile 50 mL conical tube containing 15-20 ml of ice-cold, sterile DMEM. For participants from the "Last Gift" cohort, antiretroviral drugs (200 nM raltegravir, 100 nM nevirapine, and 25 nM darunavir) were added to the collection medium. The samples were then transported overnight on ice to the UNC lab and processed within 24 hours of harvest for CNS cell isolation.

Flow cytometry analysis

To evaluate proper separation of microglia from other cell types, the positive CD11b+ fraction or TMEM119+ fraction were stained for flow cytometry analysis. For CD68 and CD45 staining of the CD11b$^+$ fractions and pre-isolated CNS cells from NHPs, the cells were fixed and permeabilized with BD Cytofix/Cytoperm (BD Biosciences). The samples were incubated with anti-CD68 (Cat#sc-20060, clone KP1, Santa Cruz Biotech), followed by FITC labeled anti-mouse secondary antibody (Cat#115-097-003, Jackson ImmunoResearch). The samples were then rinsed and incubated with anti-CD45-APC (Cat#561290, BD PharMingen,). The TMEM119+ fractions from NHP were assayed for the co-expression of CD11b and TMEM119, which were incubated with anti-TMEM119 (Cat#HPA051870, rabbit anti-TMEM119, Sigma,) followed by incubation with goat-anti-rabbit Cy3-conjugated secondary antibody (Cat#111-167-003, Jackson ImmunoResearch) and anti-CD11b-FITC (Cat#100715, Caprico Biotechnologies Inc.). The CD11b$^+$ fractions from human samples in the pre-isolation and post-isolation steps were evaluated by staining CD11b and TMEM119. The samples were incubated with the same rabbit anti-TMEM119 primary antibody, followed by incubation with FITC-conjugated anti-rabbit secondary antibody and APC-labeled anti-CD11b (Cat#4007042,

Caprico Biotechnologies Inc.). CD11b+ fractions isolated from human samples at both P0 and P2 phases were analyzed by flow cytometry of CD4 (Cat#562402, PE-CF594 labeled, BD Pharmingen™,), CXCR4 (Cat #557145, PE labeled, BD Pharmingen) and CCR5 (Cat#MAB182-100, BD Pharmingen) labeled with 488-conjugated secondary antibodies. Appropriate isotype controls were included to assess background levels of fluorescence. For the indirect staining, we included un-conjugated isotype to match the primary antibody species and isotypes, the samples were then incubated with the same labeled secondary so that the level of specific staining by the primary antibodies was accurately determined. For directly labeled primary antibodies, the isotype controls conjugated with the same labels were chosen. For indirect staining, the highly adsorbed secondary antibodies with minimal cross-reactivity among different antibody host species were carefully selected. Fluorescence was measured on either FACSCalibur (BD bioscience) or on Attune NxT (Life technologies) and analyzed with FlowJo software (Treestar, Ashland, USA).

Quantitative reverse transcription-PCR (RT-qPCR) and RT-digital droplet PCR (RT-ddPCR)

RNA was isolated using the RNAeasy Mini kit (QIAGEN), while cDNA was synthesized using the SuperScript IV First-strand synthesis system (Life Technologies) after treating RNA with DNase I. To measure NHP CD3^{+} T RNA in the isolated NHP microglia and in the BrMCs spiked with a different amount of NHP CD3^{+} T cells, RT-qPCR was performed with the NHP CD3E primer/probe (Rh01062242_m1, Thermo Fisher Scientific) and Taqman Universal PCR master mix (Thermo Fisher Scientific) on a QuantStudio5 Applied Biosystems (Thermo Fisher Scientific). RT-ddPCR was used to quantify CD3 RNA levels in the isolated human MG during the pre- and post-isolation steps. The spike experiment was performed as in NHP samples, using human CD3 primer/probe (Hs01062241_m1, Thermo Fisher Scientific) in ddPCR Supermix for Probes (Bio-Rad), performed in the QX200 AutoDG Droplet Digital PCR System (Bio-Rad) in triplicates.

Determine viral total or integrated DNA by ddPCR or Alu-PCR

Genomic DNA was extracted from BrMCs and snap-frozen brain tissues using QIAamp DNA Mini Kit (Qiagen, cat#51306). After extraction, DNA precipitation was

performed to enrich DNA. Concentrations of DNA were determined by NanoDrop One (Thermo Fisher Scientific). HIV DNA copies were quantified by ddPCR using the Bio-Rad QX200 Droplet Reader (1). Copy number was calculated as the mean of 3 replicates normalized to one million cells determined by RPP30 (total cell count) (1-3). Integrated HIV DNA was amplified with the Alu-gag PCR based on a previously published method (4) via a two-step PCR assay adapted from the method of O'Doherty et al (5). Total SIV-DNA was determined by ddPCR targeting the gag region of SIV. The primers and probe were adapted from a previous report (6): forward primer (SGAG21), 5′- GTCTGCGT CATcTGGTGCATTC-3′; reverse primer (SGAG22), 5′- CACTAGKTGTCTCTGCAC TATcTGTTTTG-3′, reverse primer (SGAG22), 5′- CACTAGKTGTCTCTGCACTATcTG TTTTG-3′.

Supernatant HIV/SIV RNA quantified by ddPCR.

Viral RNA was extracted from BrMCs/MG or BrMCs/MG culture supernatants by RNAeasy Mini kit (Qiagen) or QIAamp viral RNA mini kit (Qiagen). RNA was then treated with DNase I, and cDNA was synthesized by SuperScript IV First-strand synthesis system (Life technologies). The ddPCR mix for the RNA assay was consisted of: 11 µl 2x ddPCR™ super mix for probes (Bio-Rad); 200 nM of primers; 400 nM probe mix and 8 µl of the cDNA into a final volume of 22 µl. Two sets of HIV primers/probe annealing to gag (3) and long-LTR region (7) were used to measure HIV RNA. PCR cycling condition was 95°C 10 minutes; 45 cycles of 30 s at 94°C and 57°C for 60 s, and a final droplet cure step of 10 min at 98°C. Droplets were read and analyzed by QuantaSoft in the absolute quantification mode.

MG latency reversal and quantitative viral outgrowth assay (QVOA)

Triplicates of purified MG (10^5 cells/well) were cultured in the presence of ART (100 nM Nevirapine, 25 nM Darunavir, and 200 nM Raltegravir) for up to 2 weeks until the cells fully attached to dishes. The cell monolayers were rinsed with PBS and activated with LRAs for 7 days. The culture supernatants were harvested at days 1, 4, and 7 and assayed for cell-free viral RNA release by RT-ddPCR.

The quantitative viral outgrowth assay (MG-QVOA) was performed with 1-4 million

MG. Cells were plated in replicates with limiting dilutions of 10^6 (0-3 cultures), 10^5 (3-12 cultures), 10^4 (3-6 cultures), 10^3 (3 cultures), or 10^2 (3 cultures) per well, then activated with 250 nM SAHA plus 25 nM CM272 for 7 days. Culture supernatants were collected for de novo infection as described below. Cells were washed and co-cultured with CD8-depleted PHA blasts from an HIV-negative donor or HIV-permissive cells, Molt4/CCR5. Culture supernatants harvested at days 17, 21, 24, and 28 were assayed for viral RNA by RT-ddPCR. HIV p24 antigen was measured by p24 ELISA (ZeptoMetrix RETROtek, HIV-1 p24 Antigen ELISA, Thermo Fisher Scientific) or by SIMOA ELISA (Quanterix, Billerica, MA, USA) (8). Cultures were scored as positive if HIV RNA or HIV p24 detected at day 21 and increased at day 28. A maximum likelihood method was used to estimate the frequency of MG infection (9, 10), reported as infectious units per million microglia (IUPM).

T cell outgrowth assay (T cell bulk QVOA)

T cells outgrowth assay was performed as previously described (11, 12). Briefly, CNS T cells (10^4-10^6 cells) isolated from 20-60 grams of fresh brain tissues were plated in either a single well or in triplicate wells. Total $CD4^+$ T cells from PBMCs were isolated (StemCell Technology) which was plated in one well and stimulated with PHA/IL-2 and irradiated PBMCs from an uninfected donor for 24hrs. CD8-depleted PHA blasts were added to propagate outgrowth viruses per standard protocol. Day 8 post-stimulation, cells were mixed well, split half, and transferred to a new plate for continued viral outgrowth. Supernatants were harvested from each well on days 15, 19 and 23 and were used to determine p24 antigen by both standard ELISA and ultra-sensitive SIMOA methods (8).

De novo HIV infection in PBMCs and MG

PBMCs were depleted of $CD8^+$ T cells and maintained in RPMI1640 medium supplemented with 1% penicillin/streptomycin, 10% FBS and 100 U/mL IL-2. To stimulate the cells, 2 µg/mL PHA was added for 2-3 days before they were used for the viral outgrowth assay and de novo infection, as described previously. MG were isolated from fresh post-mortem brain tissues obtained from HIV-negative donors at NDRI. The MG were cultured for up to 2 weeks.

For the de novo infection, both PBMC PHA blasts and MG were spin-inoculated with 500 µl supernatants from HIV RNA-positive wells with LRA-stimulated MG from donor 2. Control cells were infected with supernatants from MG cultures isolated from HIV-negative donors. In the PBMC-blasts infection, ART (100 nM Nevirapine, 25 nM Darunavir, and 200 nM Raltegravir) was included as a treatment control. The supernatants were discarded the next day, and outgrowth supernatants were collected every 3-4 days for 2 weeks in cultures with PBMC blasts and up to 92 days in cultures with MG.

For the MG de novo infection, each well of cells was split into two groups at day 49 post-infection. One group served as an infection control, while the other group was treated with the CCR5 inhibitor Maraviroc (1 µM). The cells were continued to culture for several more weeks. The supernatants were assayed for HIV RNA and/or p24 antigen release by RT-ddPCR and p24 ELISA (Thermo Fisher Scientific and ZeptoMetrix RETROtek, HIV-1 p24 Antigen ELISA).

HIV *env* sequence analysis

The methods for proviral DNA from brain tissue and PBMCs and viral RNA from MGs-outgrowth virus were adapted from our previous publication (13). In Brief, nested PCR was used to amplify single genome full length *env*. DNA extracted from ante-mortem PBMCs and post-mortem tissues. RNA was extracted from day 21 culture supernatants of MG-QVOA positive wells by QIAamp viral RNA mini kit (Qiagen). RNA was treated with DNase, and cDNA was synthesized from the isolated RNA using SuperScript IV First-strand synthesis system (Life technologies). Genomic DNA or cDNA was then diluted based on ddPCR quantification data. This limited dilution PCR reaction can prevent PCR recombination, ambiguous base calls and allow the amplification of viral single genomes (14, 15). Template cDNA and HIV DNA extracted from tissues were diluted until approximately 30% of the second-round reactions were positive for the correctly-sized amplification product. Primers used for the first round were 5' FENVouter (forward) TTAGGCATCTCCTATGGCAGGAA and 3' RENVouter (reverse) TCTTAAAGGTACCTGAGGTCTGACTGG. First round PCRs were performed using the Advantage 2 PCR Kit from Takara (Takara, cat# 639206) following manufacturer's

recommendations using the 10X SA Buffer. Cycling conditions were 95 °C for 1 min, 35 cycles of 95 °C 15 sec, 57 °C 30 sec, 68 °C 3 min with a final extension at 68 °C for 10 min. The second round PCR were done using 5'FENVinner: GAGCAGAAGACAGTGGC AATGA (forward) and 3'RENVinner: CCACTTGCCACCCATBTTATAGCA (reverse). Cycling conditions were 95 °C for 1 min, 30 cycles of 95 °C 15 sec, 64 °C 30 sec, 68 °C 3 min with a final extension at 68 °C for 10 min. PCR clean ups were done on the second-round reaction products using QIAquick PCR Purification Kit (cat# 28106). DNA was quantified using Qubit dsDNA HS Assay Kit (cat#Q32854, Invitrogen). Quality and integrity were measured using Genomic DNA Screen Tape in combination with the 2200 TapeStation System (cat#5067-5366, Genomic DNA Reagents; Cat#5067-5365, Genomic DNA Screen Tape).

Single Genome amplicons were prepared for deep sequencing using the Nextera XT DNA Library Preparation Kit (Illumina FC-131-1096) with indexing of 96-samples per run (Nextera XT index kit set A FC-131-2001) per manufacturer's protocols. To assembly of full-length HIV env sequences, we used our designed pipeline to recover full-length env HIV sequences from the paired end reads (13). The pipeline included a preliminary step of quality control, which included trimming reads for PHRED quality above or equal 30 and removal of Illumina adapters. Next, overlapping identical paired forward and reverse reads were merged and pre-mapped to HXB2 reference genome. Cleaned reads were re-mapped to the de novo assembled near full-length *env* sequence before generating the final consensus sequence. The minimum acceptable coverage was set to 10,000 reads. To identify mixtures (ie. suggesting multiple amplified HIV templates), we screened all generated full-length *env* contigs. Mixtures were identified based on read coverage and variant calling. Contigs with evidence of single nucleotide polymorphisms (SNPs) with a frequency >1% were considered mixtures and excluded from further analyses. To identify defective or hypermutant sequences, full-length envelope containing large deletions (>100bp) were considered defective (16, 17). Deleterious stop codons were identified using the Gene Cutter tool (Los Alamos HIV Database). Any contigs containing a stop codon were considered defective. No hyper mutated *env* were found using the Los Alamos HIV Database Hypermut 2 program with the participant's consensus sequence (18, 19). Proviruses with a number of mutations significantly higher than the participant's

consensus (Fisher exact test $p < 0.05$) were considered hyper-mutant and were not included in the downstream analyses described below. Maximum-likelihood phylogeny reconstruction of intact full-length env sequences was performed with IQtree (20) and visualized with ggtree R package (21). Viral diversity was defined as the average pairwise genetic distance between sequences from a compartment using the Tamura Nei 93 (TN93) correction for multiple hits (22). Viral divergence was assessed by computing the mean pairwise distance (TN93) between viral populations sampled across anatomical sites. Viral tropism of each variant was inferred from the V3 amino acid sequence using geno2pheno (23). We applied a conservative 10% false positive rate threshold for co-receptor CXCR4 usage based on recommendation from the European Consensus Group on clinical management of HIV-1 tropism testing. Visualization of the amino-acid variation of the V3 region was done using ggmsa R package (24). Sequences are available upon reasonable request.

Near full-length MG-OGV genome sequencing

Near full-length sequencing of HIV induced from MG and HIV OGV was performed as previously described (25, 26) through amplification by nested PCR of overlapping 5' and 3' half-genomes. Viral RNA was extracted from culture supernatant using Qiagen BioRobot EZ1 Workstation with EZ1 Virus Mini kit (Qiagen) and immediately employed as template to generate cDNA via Superscript III (Life Technologies) reverse transcriptase system in two separate reactions using B5R1 primer to generate 5' half cDNA and R3B3R to generate 3' half cDNA. Limited dilution PCR was then performed, as described, through separate amplification by nested PCR of overlapping 5' and 3' half-genomes. For 5' half-genome, we used forward primer U5.B1.F (5' CCTTGAGTGCTTCAAGTAGTGTGTGCCCG TCTGT-3') and reverse primer B5R1 (CTTGCCACACAATCATCACCTGCCAT) for first round of amplification followed by forward primer U5.B4.F (5'-GTAGTGTGTGCCCGTC TGTTGTGTGACTC-3') and reverse primer B5R2 (5'-CAATCATCACCTGCCATCT GTTTTCCATA-3') for second round of amplification. For 3' half genome, we used forward primer B3F1 (5'-ACAGCAGTACAAATGGCAGTATT-3') and reverse primer R3B3R (5'-ACTACTTGAAGCACTCAAGGCAAGCTTTATTG-3') for first round followed by forward

primer B3F3 (5'-TGGAAAGGTGAAGGGGCAGTAGTAATAC-3’) and reverse primer R3B6R (5’-TGAAGCACTCAAGGCAAGCTTTATTGAGGC-3’). The 5’ round two primers U5.B4F and B5R2 flank the 5’ end of LTR U5 segment (HXB2 nt 552-581) and the 3’ end of pol (HXB2 nt 5,040-5,068), respectively, while the 3’ round two primers B3F3 and R3B6R flank the 3’ end of pol (HXB2 nt 4,956-4,983) and 3’ end of LTR R segment (HXB2 nt 9,607-9,636), respectively. The resulting ~5kb fragments overlap at pol and together contain nearly the entire genome, aside from the nucleotides complementary to the U5.B4F and R3B6R primers, some of which may be derived from the primer sequence rather than the viral sequence. For each half-genome, 4-10 amplicons were generated, which were sequenced via MiSeq platform (Illumina). Raw paired reads were aligned to HXB2 genome (GenBank: K03455.1) with 75% quality threshold to generate consensus sequences. Sequences with ambiguities were discarded from analysis. These consensus sequences were combined at the overlapping pol region and major genes (gag, pol, vif, vpu, tat, rev, env, nef) were inspected to ensure intact reading frames. Alignments and sequences analysis were performed using Geneious Prime software. Sequences were deposited in GenBank, and the accession numbers of these sequences are OQ325479 (HIV induced from MG) and OQ325480 (HIV from MG OGV).

Viral tropism assays

Previously described protocols (27) were used to amplify *envs* from MG and PBMC outgrowth cultures. Briefly, RNA was extracted from outgrowth cultures, converted to cDNA and full-length *env* genes were PCR amplified at limiting dilution. Proviral *envs* from PBMCs and the basal ganglia were commercially synthesized. Full-length *env* genes were cloned into pcDNA3.1D/V5-His-TOPO expression vector (Invitrogen) using the pcDNA3.1 TOPO expression kit (Invitrogen). Clones were then sequenced to confirm identity to the amplified sequence and used to generate pseudotyped luciferase reporter viruses (27). Affinofile cells can be induced to express different levels of CD4 and CCR5 (28). In this study, M-tropism was determined based on the ability of viruses to enter cells expressing low CD4/high CCR5 relative to their ability to infect cells expressing high CD4/high CCR5 (29). M- and T cell-tropic controls were previously described (27, 30).

Supplementary Figure legends

Figure S1. Isolation of BrMCs from NHPs (Related to Figure 1A). The representative FACS plots showed CD68+ BrMCs in the CNS single cell suspension before the isolation step (**A**) and after CD11b selection (**B**). CD45 staining distinguished MG ($CD45^{low}$CD68+ MG) from other brain myeloid cells ($CD45^{high}$CD68+ macrophages)(**D**). (**C**) The representative image showed isolated BrMCs attached to the culture surface 2 weeks post-culture ex vivo, which expressed BrMC marker CD68 (green). Nuclei were shown as blue. Scale bar, 250 μm.

Figure S2. FACS gating strategy to assess isolation efficiency and MG marker expression (Related to Figure 1C). Size and granularity gating on the intact single cell (**A**) and gating on singlets (**B**) were shown. The gating of the negative control (**C**) was shown in which the samples were incubated with un-conjugated Rabbit IgG isotype, followed by Cy3 anti-rabbit secondary antibody and mouse IgG1-FITC isotype. Fluorescence minus one (FMO) control 1 (**D**) stained with anti-CD11b FITC, and FMO control 2 (**E**) stained with anti-TMEM119 rabbit antibody and Cy3 conjugated anti-rabbit secondary antibody, were shown. (**F-G**) The histograms showed the matched isotype controls (in blue) compared with the specific antibodies (in red).

Figure S3. A pilot screen assay showed HDACi inhibitor SAHA was the most potent LRA to induce HIV expression in the latent microglia model (Related to Figure 4B). A microglia model of HIV latency (From Dr. Brandon K. Harvey group in NIH) was treated with the following LRAs for 2 days: HDACi SAHA (500 nM); the canonical NF-kB agonists PEP005 (12 nM), Byrostatin (5 nM); Prostratin (5 μM); histone methyltransferase inhibitor GSK343 (4 μM); DNA methylation inhibitor, 5-AZA-2'-deoxycytidine (AZA-dC, 10 μM); and crotonylation inducer NaCr (40 mM). The cells were then collected for total RNA extraction and RT-qPCR for HIV expression after normalized to β-actin. ****, $p<0.0001$, compared with control treatment by one-way ANOVA (n=3).

1. Strain MC, Lada SM, Luong T, Rought SE, Gianella S, Terry VH, et al. Highly precise measurement of HIV DNA by droplet digital PCR. *PLoS ONE.* 2013;8(4).
2. Pinheiro LB, Coleman VA, Hindson CM, Herrmann J, Hindson BJ, Bhat S, et al. Evaluation of a droplet digital polymerase chain reaction format for DNA copy number quantification. *Anal Chem.* 2012;84(2):1003-11.
3. Malnati MS, Scarlatti G, Gatto F, Salvatori F, Cassina G, Rutigliano T, et al. A universal real-time PCR assay for the quantification of group-M HIV-1 proviral load. *Nat Protoc.* 2008;3(7):1240-8.
4. Liszewski MK, Yu JJ, and O'Doherty U. Detecting HIV-1 integration by repetitive-sampling Alu-gag PCR. *Methods.* 2009;47(4):254-60.
5. Mavigner M, Lee ST, Habib J, Robinson C, Silvestri G, O'Doherty U, et al. Quantifying integrated SIV-DNA by repetitive-sampling Alu-gag PCR. *J Virus Erad.* 2016;2(4):219-26.
6. Cline AN, Bess JW, Piatak M, Jr., and Lifson JD. Highly sensitive SIV plasma viral load assay: practical considerations, realistic performance expectations, and application to reverse engineering of vaccines for AIDS. *J Med Primatol.* 2005;34(5-6):303-12.
7. Yukl SA, Kaiser P, Kim P, Telwatte S, Joshi SK, Vu M, et al. HIV latency in isolated patient CD4(+) T cells may be due to blocks in HIV transcriptional elongation, completion, and splicing. *Sci Transl Med.* 2018;10(430).
8. Stuelke EL, James KS, Kirchherr JL, Allard B, Baker C, Kuruc JD, et al. Measuring the Inducible, Replication-Competent HIV Reservoir Using an Ultra-Sensitive p24 Readout, the Digital ELISA Viral Outgrowth Assay. *Front Immunol.* 2020;11:1971.
9. Trumble IM, Allmon AG, Archin NM, Rigdon J, Francis O, Baldoni PL, et al. SLDAssay: A software package and web tool for analyzing limiting dilution assays. *J Immunol Methods.* 2017;450:10-6.
10. Myers LE, McQuay LJ, and Hollinger FB. Dilution assay statistics. *J Clin Microbiol.* 1994;32(3):732-9.
11. Archin NM, Kirchherr JL, Sung JA, Clutton G, Sholtis K, Xu Y, et al. Interval dosing with the HDAC inhibitor vorinostat effectively reverses HIV latency. *J Clin Invest.* 2017;127(8):3126-35.
12. Crooks AM, Bateson R, Cope AB, Dahl NP, Griggs MK, Kuruc JD, et al. Precise Quantitation of the Latent HIV-1 Reservoir: Implications for Eradication Strategies. *J Infect Dis.* 2015;212(9):1361-5.
13. Chaillon A, Gianella S, Dellicour S, Rawlings SA, Schlub TE, De Oliveira MF, et al. HIV persists throughout deep tissues with repopulation from multiple anatomical sources. *J Clin Invest.* 2020;130(4):1699-712.
14. Bruner KM, Murray AJ, Pollack RA, Soliman MG, Laskey SB, Capoferri AA, et al. Defective proviruses rapidly accumulate during acute HIV-1 infection. *Nat Med.* 2016;22(9):1043-9.
15. Ho YC, Shan L, Hosmane NN, Wang J, Laskey SB, Rosenbloom DI, et al. Replication-competent noninduced proviruses in the latent reservoir increase barrier to HIV-1 cure. *Cell.* 2013;155(3):540-51.
16. Hiener B, Horsburgh BA, Eden JS, Barton K, Schlub TE, Lee E, et al. Identification of Genetically Intact HIV-1 Proviruses in Specific CD4(+) T Cells from Effectively Treated Participants. *Cell Rep.* 2017;21(3):813-22.
17. Pinzone MR, VanBelzen DJ, Weissman S, Bertuccio MP, Cannon L, Venanzi-Rullo E, et al. Longitudinal HIV sequencing reveals reservoir expression leading to decay which is obscured by clonal expansion. *Nature communications.* 2019;10(1):728.
18. Rose PP, and Korber BT. Detecting hypermutations in viral sequences with an emphasis on G --> A hypermutation. *Bioinformatics (Oxford, England).* 2000;16(4):400-1.
19. Los Alamos National Laboratory, and National Institutes of Health. HIV databases. http://www.hiv.lanl.gov/. 2019.

20. Schmidt HA, Minh BQ, von Haeseler A, and Nguyen L-T. IQ-TREE: A Fast and Effective Stochastic Algorithm for Estimating Maximum-Likelihood Phylogenies. *Molecular Biology and Evolution.* 2014;32(1):268-74.
21. Yu G, Smith DK, Zhu H, Guan Y, and Lam TT-Y. ggtree: an r package for visualization and annotation of phylogenetic trees with their covariates and other associated data. *Methods in Ecology and Evolution.* 2017;8(1):28-36.
22. Tamura K, and Nei M. Estimation of the number of nucleotide substitutions in the control region of mitochondrial DNA in humans and chimpanzees. *Molecular biology and evolution.* 1993;10(3):512-26.
23. Beerenwinkel N, Däumer M, Oette M, Korn K, Hoffmann D, Kaiser R, et al. Geno2pheno: Estimating phenotypic drug resistance from HIV-1 genotypes. *Nucleic Acids Res.* 2003;31(13):3850-5.
24. Zhou L, Feng T, Xu S, Gao F, Lam TT, Wang Q, et al. ggmsa: a visual exploration tool for multiple sequence alignment and associated data. *Briefings in Bioinformatics.* 2022;23(4).
25. Keele BF, Giorgi EE, Salazar-Gonzalez JF, Decker JM, Pham KT, Salazar MG, et al. Identification and characterization of transmitted and early founder virus envelopes in primary HIV-1 infection. *Proc Natl Acad Sci U S A.* 2008;105(21):7552-7.
26. Salazar-Gonzalez JF, Salazar MG, Keele BF, Learn GH, Giorgi EE, Li H, et al. Genetic identity, biological phenotype, and evolutionary pathways of transmitted/founder viruses in acute and early HIV-1 infection. *J Exp Med.* 2009;206(6):1273-89.
27. Schnell G, Joseph S, Spudich S, Price RW, and Swanstrom R. HIV-1 replication in the central nervous system occurs in two distinct cell types. *PLoS Pathog.* 2011;7(10):e1002286.
28. Johnston SH, Lobritz MA, Nguyen S, Lassen K, Delair S, Posta F, et al. A quantitative affinity-profiling system that reveals distinct CD4/CCR5 usage patterns among human immunodeficiency virus type 1 and simian immunodeficiency virus strains. *J Virol.* 2009;83(21):11016-26.
29. Joseph SB, Lee B, and Swanstrom R. Affinofile Assay for Identifying Macrophage-Tropic HIV-1. *Bio Protoc.* 2014;4(14).
30. Joseph SB, Arrildt KT, Swanstrom AE, Schnell G, Lee B, Hoxie JA, et al. Quantification of entry phenotypes of macrophage-tropic HIV-1 across a wide range of CD4 densities. *J Virol.* 2014;88(4):1858-69.

Supplementary Figure 1 (Related to Figure 1)

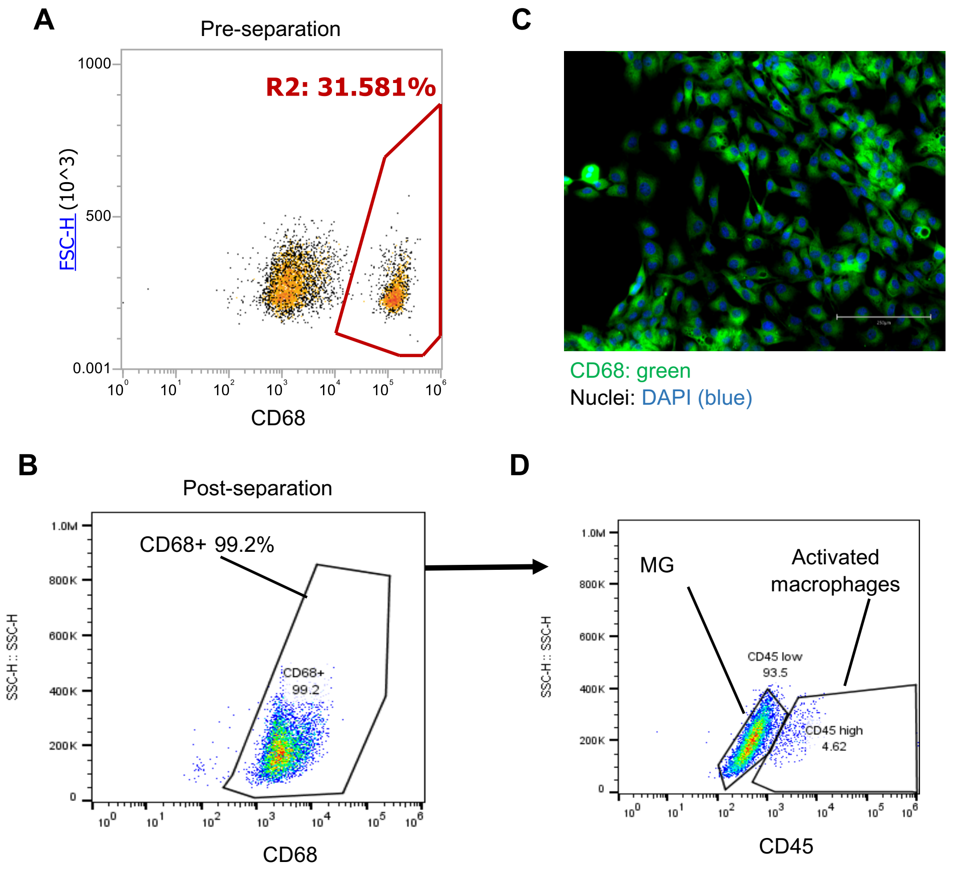

Supplementary Figure 2 (Related to Figure 1C)

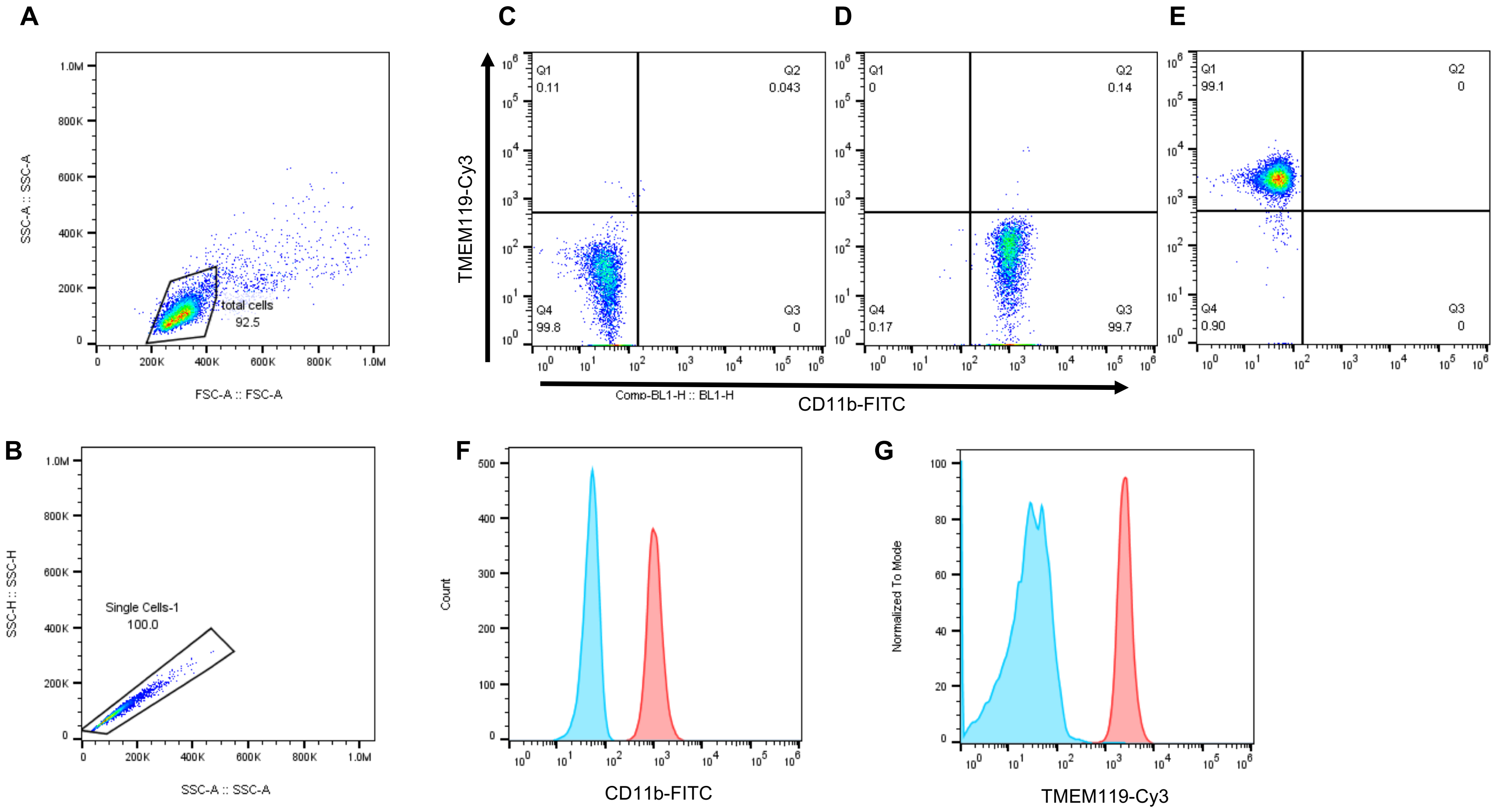

Supplementary Figure 3 **(related to Figure 4B)**

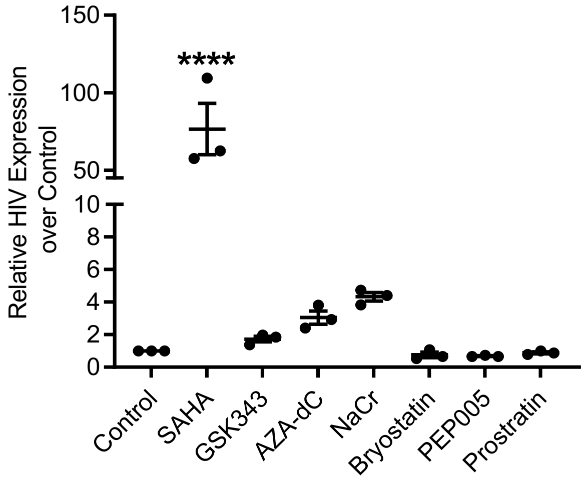

Supplementary Table 1. Animals information in this study (Related to Figure 1).

Animal #	Species	Sex	Age at infection (years)	Peak viremia (copies/mL)	Peak viremia, Pre-ART (copies/mL)	CD4 Count at NX (cells/µL)	Plasma VL at NX (copies/mL)
1	Rhesus macaque	Male	4.04	5.80E+07	1.43E+05	988	<60
2	Rhesus macaque	Male	3.00	2.36E+07	1.01E+06	868	<60
3	Rhesus macaque	Male	4.04	3.38E+07	4.80E+06	755	<60
4	Rhesus macaque	Male	3.19	8.19E+06	4.98E+06	924	<60

NX, Necropsy

Supplementary Table 2. Summary of clinical variables of HIV negative brain donors (Related to Figure 2).

NDRI #	Age of death	Gender	Terminal disease	Race
N3	70	F	Cardiac Arrest	Caucasian
N4	88	F	Cardiac Arrest	Caucasian
N5	48	M	Cardiac Arrest	Caucasian
N6	88	M	Cardiogenic Pulmonary Edema	Caucasian

Gender abbreviations: F, Female; M, male.

Supplementary Table 3. MG-QVOA of donor 2 (Related to Figure 4D-E).

		HIV RNA (copies/mL)			HIV p24(ng/mL)		
# BrMC	Replicate	Positive [a]	Mean [b]	Range [b]	Positive [c]	Mean [d]	Range [d]
100,000	3	3	2.9E+06	Max 3.48E+6, Min 2.0E+1	1	465	Max 1395,Min 0
10,000	6	3	1.09E+02	Max 1.69E+2, Min 0	0	0	Max 0, Min 0
1000	3	1	5.4E+01	Max 1.62E+2, Min 0	0	0	Max 0, Min 0
100	3	1	5.17E+01	Max 1.55E+1, Min 0	0	0	Max 0, Min 0
0	3	0	0	Max 0, Min 0	0	0	Max 0, Min 0

Mean likelihood estimate (MLE) of HIV RNA IUPM for donor 2 was 111 per million, and Exact confidence interval (CI) was 42.6, 252.1.Traditional IUPM(p24) was 3.57, and Exact confidence interval (CI) was 0.17, 17.6.

[a] HIV gag RNA positive wells were identified at day 21 and further confirmed at day 28.[b] values shown are means and ranges of HIV gag RNA (copies/mL) in the supernatants at day 21 of each dilution. [c] p24 positive wells were identified at day 21 and further confirmed at day 28.[d] values shown are means and ranges of p24 in the supernatants at day 21 of each dilution.

Supplementary Table 4. MG-QVOA of donor 1 (Related to Figure 4D-E).

		HIV RNA (copies/mL)			HIV p24(pg/mL)		
**# BrMC**	**Replicate**	**Positive [a]**	**Mean [b]**	**Range [b]**	**Positive**	**Mean [d]**	**Range [d]**
1,000,000	3	2	1.13E+02	Max 2.07E+2, Min 0	1	3.2	Max 9.6,Min 0
100,000	3	0	0	Max 0, Min 0	0	0	Max 0, Min 0
10,000	3	0	0	Max 0 Min 0	0	0	Max 0, Min 0
1000	3	0	0	Max 0, Min 0	0	0	Max 0, Min 0
0	3	0	0	Max 0, Min 0	0	0	Max 0, Min 0

Mean likelihood estimate (MLE) of IUPM for donor 1 was 0.96 per million, and Exact confidence interval (CI) was 0.145, 3.65. Traditional IUPM(p24) was 0.36, and Exact confidence interval (CI) was 0.017, 1.76.

[a] HIV gag RNA positive wells and [c] HIV p24 positive wells were identified at day 21 and further confirmed at day 28.The means and ranges of HIV gag RNA[b] and p24[d] antigen in the supernatants at day 21 of each dilution.

Supplementary Table 5. Microglia-QVOA results of donor 3 (Related to Figure 4D-E).

		HIV RNA (copies/mL)			HIV p24(pg/mL)		
**# BrMC**	**Replicate**	**Positive [a]**	**Mean [b]**	**Range [b]**	**Positive [c]**	**Mean [d]**	**Range [d]**
100,000	3	2	472	Max 1330, Min 0	1	<10	detectable, Min 0
10,000	3	2	20027	Max 71047, Min 0	0	0	Max 0, Min 0
1000	3	2	157	Max 608, Min 0	0	0	Max 0, Min 0
100	2	1	158	Max 528, Min 0	0	0	Max 0, Min 0
0	3	0	0	Max 0, Min 0	0	0	Max 0, Min 0

IUPM mean likelihood (MLE) estimator : 41.5 Exact confidence interval (CI): 16.1, 90.4. Traditional IUPM (p24) was 3.57, and Exact confidence interval (CI) was 0.17, 17.6. [a,c] HIV gag RNA and p24 positive wells were identified at day19 and further confirmed at day 28. [b,d] values were means and ranges in the supernatants at day 19 of each dilution.

Supplementary Table 6. MG-QVOA results of donor 4 (Related to Figure 4D-E).

		HIV RNA (copies/mL)			HIV p24(pg/mL)		
**# BrMC**	**Replicate**	**Positive [a]**	**Mean [b]**	**Range [b]**	**Positive [c]**	**Mean [d]**	**Range [d]**
100000	3	1	36	Max 109, Min 0	0	0	Max 0, Min 0
10000	3	2	113	Max 214, Min 0	1	<10	detectable, Min 0
1000	3	1	71	Max 212, Min 0	0	0	Max 0, Min 0
100	3	0	0	Max 0, Min 0	0	0	Max 0, Min 0
0	3	0	0	Max 0, Min 0	0	0	Max 0, Min 0

IUPM mean likelihood (MLE) estimator : 15.37 Exact confidence interval (CI): 4.52, 38.3. [a] HIV gag RNA positive wells were identified at day 21 and further confirmed at day 28. Traditional IUPM (p24) was 3.0, and Exact confidence interval (CI) was 0.16, 11.47.[a,c] HIV gag RNA and p24 positive wells were identified at day 21 and further confirmed at day 28. [b,d] values were means and ranges in the supernatants at day 21 of each dilution.
